# Exploiting Coordination Isomerism for Controlled Self‐Assembly

**DOI:** 10.1002/anie.201908002

**Published:** 2019-09-09

**Authors:** Nils Bäumer, Kalathil K. Kartha, Naveen Kumar Allampally, Shiki Yagai, Rodrigo Q. Albuquerque, Gustavo Fernández

**Affiliations:** ^1^ Organisch-Chemisches Institut Westfälische Wilhelms-Universität Münster Corrensstraße 40 48149 Münster Germany; ^2^ Institut für Organische Chemie Universität Würzburg Am Hubland 97074 Würzburg Germany; ^3^ Department of Applied Chemistry and Biotechnology Graduate School of Engineering Chiba University 1–33-Yayoi-cho Inage-Ku Chiba 263-8522 Japan

**Keywords:** coordination isomerism, photoresponsive behavior, self-assembly, supramolecular polymers, π-conjugated systems

## Abstract

We exploited the inherent geometrical isomerism of a Pt^II^ complex as a new tool to control supramolecular assembly processes. UV irradiation and careful selection of solvent, temperature, and concentration leads to tunable coordination isomerism, which in turn allows fully reversible switching between two distinct aggregate species (1D fibers↔2D lamellae) with different photoresponsive behavior. Our findings not only broaden the scope of coordination isomerism, but also open up exciting possibilities for the development of novel stimuli‐responsive nanomaterials.

## Graphical abstract

**Size matters**: Coordination isomerism was exploited for the first time to control the morphology of a supramolecular polymer. These observations pave the way for novel stimuli‐responsive materials and offer a new approach for size control in self‐assembled systems.

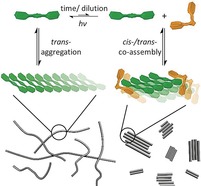

The occurrence of geometrical isomerism in coordination complexes, sometimes termed coordination isomerism, has been recognized for more than a century, and it is a commonly observed phenomenon in the photochemistry of square‐planar complexes.^1^, ^2^ In particular, Pt^II^ compounds have been reported to undergo geometrical isomerization upon UV irradiation, leading to photostationary states whose isomer composition primarily depends on the choice of ligands and solvent.^2^, ^3^ To date, geometrical isomerization of Pt^II^ complexes has been exclusively investigated at the molecular level, for instance to obtain otherwise inaccessible coordination compounds,^4^ rotors,^5^ and photoactivated catalysts.^6^

In an attempt to broaden the scope of coordination isomerism, we reasoned that the inherently different geometry of *cis* and *trans* Pt^II^ complexes might be exploited as a new method to control self‐assembly processes. Based on the versatility of metal coordination in providing multiple directional interactions, this strategy would complement the existing arsenal of tools in stimuli‐responsive materials^7^ and living supramolecular polymerization.^8^

In order to facilitate geometrical isomerism in Pt^II^ complexes, the use of small and/or conformationally unrestricted coordinating ligands appears to be a prerequisite.^9^, ^10^ Otherwise, steric repulsion between *cis*‐coordinated ligands, along with the stronger aggregation propensity of the more preorganized *trans* species, will preferentially stabilize the *trans* form, which can inhibit isomerization,^11^ or even induce photodecomposition.^12^

While screening our library of ligands, we noticed that the inclusion of an azobenzene moiety in the molecular design enhances the conformational freedom of the system,^13^, ^14^ which might allow a good balance between isomerization and aggregation. Additionally, complexation with Pt^II^ inactivates the azobenzene moiety to light irradiation so it will not influence the coordination isomerism through additional isomerization possibilities.^15^, ^16^ On this basis, we designed a new Pt^II^L_2_Cl_2_ complex (**C_1_**), with L being a 4‐phenylazopyridyl‐based ligand featuring peripheral amide groups and dodecyloxy side chains^13^, ^14^ (Scheme 1; for synthesis and characterization, see the Supporting Information). This rational choice of ligand, solubilizing groups, and hydrogen‐bonding units enables simultaneous control over coordination isomerism and self‐assembly for the first time. UV irradiation and appropriate choice of solvent, temperature, and concentration allows fully reversible switching in the aggregate morphology (1D↔2D) and represents an innovative strategy towards stimuli‐responsive self‐assembled materials.

**Scheme 1:**
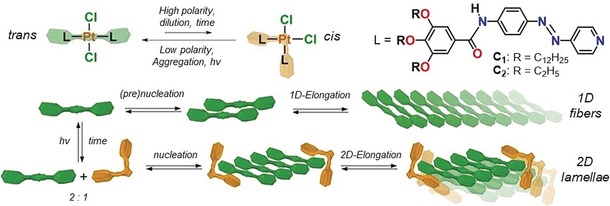
Molecular structures of **C_1_** and **C_2_**, and a cartoon representation of the supramolecular assembly of **C_1_** triggered by coordination isomerism.

The self‐assembly behavior of **C_1_**, synthesized as a pure *trans* form, was initially probed in methylcyclohexane (MCH) using variable‐temperature (VT) UV/Vis studies at 2×10^−5^ m. These experiments showed only negligible absorption changes when monomer solutions where cooled from 363 K to 293 K (Figure 1 a). However, further cooling to 273 K causes a marked red shift in the absorption maximum from 406 nm to 418 nm along with an isosbestic point at 401 nm and a concurrent hyperchromism (Figure 1 a). These spectral changes, which are independent of the cooling rate (see Figure 1 a and Figure S6), can be attributed to the aggregation of **C_1_**. Notably, the corresponding plots of absorption versus temperature monitored at different wavelengths show a rather unusual two‐step curve (Figure 1 a inset and Figure S7): a smooth regime between 363 K and around 293 K followed by a sharp transition below a critical elongation temperature (*T*_e_≈293 K) that is characteristic of a nucleated supramolecular polymerization (for thermodynamic analysis, see Figure S8 and Table S1). The initial transition, which cannot be fitted to any of the existing thermodynamic models for supramolecular polymerization, suggests a pre‐nucleation event involving conformational changes of the azobenzene group(s), such as planarization, at higher temperatures. VT dynamic light scattering (DLS) studies at temperatures above the *T*_e_ showed no significant changes in the correlation and size distribution functions (Figure S9), thus validating our hypothesis. Further cooling to 283 K (below the *T*_e_) does initiate the self‐assembly of **C_1_**, as evident by the marked increase in the particle size (Figure S9). Atomic force microscopy (AFM) on highly‐oriented pyrolytic graphite (HOPG) revealed the absence of assemblies above 293 K (Figure S10). At the *T*_e_, short rods with a uniform height of 2–3 nm and a length of 40–70 nm are observed (Figure 1 b and Figure S11), which further grow longitudinally into fibers with lengths between 60 and 700 nm (average length (*l*_ave_)=261±73 nm) when the temperature is decreased to 273 K (Figure 1 c, and Figures S12, S13). Combined 1D and 2D NMR studies, both in CDCl_3_ and MCH‐d_14_ (Figures S14–S17), demonstrate a slipped molecular packing stabilized by aromatic and N−H⋅⋅⋅Cl interactions.11b, 13 This proposed arrangement is in agreement with the packing observed in the crystal state for structurally related model compound **C_2_** with shorter ethoxy chains, which exhibits N−H⋅⋅⋅Cl, C(aromatic)−H⋅⋅⋅Cl and aromatic interactions (Figure 1 d and Figure S18).

**Figure 1:**
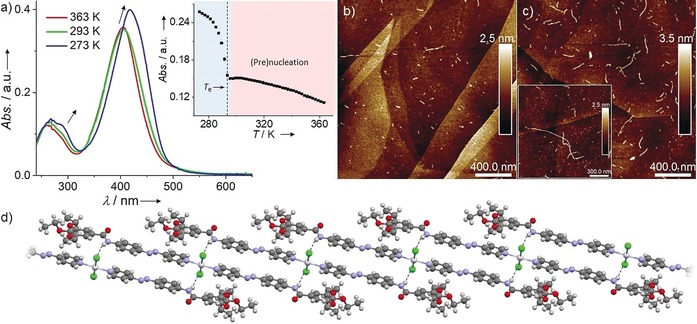
a) VT UV/Vis spectra of **C_1_** (MCH, 2×10^−5^ m, 363 K–273 K; 1 K min^−1^). Inset: plot of absorbance versus *T* extracted from VT UV/Vis (*λ*=450 nm). b, c) AFM height images recorded upon spin‐coating a 5×10^−5^ m solution of **C_1_** in MCH on HOPG at 293 K (b) and 273 K (c). d) Packing of **C_2_**, derived from X‐ray crystal analysis.

After detailed self‐assembly studies of **C_1_**, we confirmed that the azobenzene moieties are indeed inactive under UV irradiation when coordinated to Pt^II^ (Figure S19–S25).^15^ In a recent example, Shionoya and co‐workers^5^ elegantly showed that discrete Pt^II^‐centred azaphosphatriptycene molecular gears efficiently undergo coordination isomerism under irradiation in appropriate solvents. Polar solvents favour efficient *trans*‐to‐*cis* conversion due to preferential stabilization of the dipole moment of the *cis* form.^3^, ^5^ On a similar basis, we tested whether coordination isomerism is also possible for our system (**C_1_**). To our satisfaction, a new set of signals corresponding to *cis*‐**C_1_** are observed over time in the ^1^H NMR spectra when solutions of *trans*‐**C_1_** are kept under ambient conditions in moderately polar solvents such as CDCl_3_ and CD_2_Cl_2_ (Figure 2). A similar trend is observed when a concentrated solution of *trans*‐**C_1_** in CDCl_3_ (20 mm) is diluted to 1 mm (Figure S26). For both time‐ and concentration‐dependent ^1^H NMR experiments in CDCl_3_, a maximum of 33 % *cis*‐**C_1_** is obtained at equilibrium. The ratio of *cis*‐**C_1_** can be further increased to 40 % in more polar solvents such as DMSO using high temperatures (Figure S27). However, the strong hydrophobicity of **C_1_** due to the presence of long alkyl chains results in rapid precipitation even when using these harsh conditions, which precludes further analysis in polar media. Nevertheless, using the same experimental protocol for less hydrophobic **C_2_** allowed us to achieve a maximum *cis* ratio of 73 % (Figure S28). Decreasing the solvent polarity by using CD_2_Cl_2_ leads to a reduction in the maximum amount of formed *cis*‐**C_1_** (10 %), whereas no traces of *cis*‐**C_1_** were observed in nonpolar solvents such as MCH‐d_14_ and TCE‐d_2_ (*c*=1×10^−3^ m, Figure 2 and Figures S29, S30). This behavior can be rationalized by comparing the relative stability of both isomers using DFT calculations (Figure S31). Thus, while polar and dilute solutions stabilize *cis*‐**C_1_**, high concentration and solvents of low polarity favor the *trans* form, a phenomenon that appears to be reinforced by aggregation. Accordingly, we expect that only *trans*‐**C_1_** has the appropriate geometry to promote aggregation, rendering the distorted *cis* form as a dormant species. Interestingly, the reverse *cis*‐to‐*trans* isomerization of **C_1_** can be readily achieved by UV irradiation, irrespective of the solvent polarity (CDCl_3_ 33 % to 16 % and DCM‐d_2_ 10 % to 2 %; Figure 2 and Figure S32). The absence of the free ligand in solution during this transition is indicative of a twisting mechanism as the most probable isomerization pathway.^10^ Even though UV irradiation does not fully back‐isomerize the system, a complete recovery of *trans*‐**C_1_** is possible by re‐dissolution of the corresponding *cis*‐containing mixtures in a nonpolar solvent (MCH) upon evaporation of the polar solvent (CDCl_3_) at high concentration (Figure S33).

**Figure 2:**
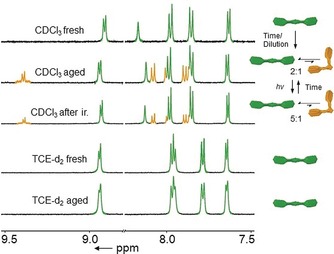
^1^H NMR spectra of **C_1_** in CDCl_3_ showing the time‐dependent formation of *cis*‐**C_1_** and the reversible back‐isomerization upon UV irradiation (*λ=*365 nm) (top). ^1^H NMR of **C_1_** in TCE‐d_4_ showing no isomerization over time (bottom; *c*=1×10^−3^ m, *T*=298 K).

We envisaged that this precise control over the coordination isomerism of **C_1_** could represent an efficient method to tune supramolecular assembly processes. To this end, a small volume (30 μL) of an equilibrated mixture of *trans*‐**C_1_** (67 %) and *cis*‐**C_1_** (33 %) at high concentration (1 mm) in chloroform was rapidly dried using argon flow to avoid back‐isomerization. The resulting fine powder was immediately thereafter dissolved in MCH (3 mL) to a final concentration of 1×10^−5^ m (100‐fold lower than in NMR experiments, in which back isomerization was observed), heated to 363 K and finally subjected to VT UV/Vis experiments. Upon cooling from 363 K to 283 K, small fluctuations in the absorption without a clear trend were observed, which can be attributed to a weak coupling of the π‐scaffolds (Figure S34). Further cooling to 273 K leads to a bathochromic shift, which is a distinctive spectral feature of slipped aggregate formation. However, this spectrum differs from the one obtained for pure *trans*‐**C_1_** (Figure 3 a), thus indicating that a different self‐assembly pathway occurs in the mixture of isomers. Analysis of the VT cooling curves reveals a lower *T_e_* for the mixture of *cis*+*trans*‐**C_1_** compared to the pure *trans*‐**C_1_** species (Figure 3 a inset, Figure S36). This delayed aggregation process (>4 h for the mixture versus <10 min for pure *trans*) is also evident from kinetic UV/Vis studies (Figure S37). VT ^1^H NMR also supports the dormant nature of the *cis*‐**C_1_** isomer, since the critical aggregation concentration of the *cis*+*trans* mixture is around 2.5 times higher (*c*=2.5×10^−3^ m) than that of the pure *trans*‐**C_1_** in CDCl_3_. Notably, all proton signals from *trans*‐**C_1_** in the mixture followed the same trend as for the compound in isolation (Figure 3 d and Figure S38), thus suggesting a similar molecular packing. In contrast, most signals of the *cis*‐**C_1_** species in the mixture undergo no broadening and less pronounced shifts upon cooling (Figure 3 d and Figure S38). In particular, the fact that some protons, for example, the amide protons H′_e_ are slightly deshielded (Figure 3 d, orange signals at ca. 8 ppm) suggests that these groups might add as stoppers to the active ends of the supramolecular fibers. This attenuated growth is further supported by dispersion‐corrected PM6 simulations, which reveal a significantly lower stability for a hexamer containing two *cis* isomers instead of pure *trans* due to the loss of intermolecular interactions. Further, the simulations reveal that co‐assembly with the *cis* isomer disrupts the alkyl chain shell around the stacked aromatic units of pure *trans*‐**C_1_** (Figure 3 e and Figures S39–S41). VT DLS experiments in MCH (*c*=2×10^−4^ m) yield considerably smaller particle sizes for the *cis*+*trans* mixture compared to the pure *trans* species under identical conditions (maxima at 170 nm versus 2600 nm; Figure S42). AFM measurements at 2×10^−5^ m demonstrate the formation of short rigid rods (Figure 3 c) at the *T*_e_ (283 K) with a uniform height of 2 nm and lengths between 30 and 110 nm (*l*_ave_=48.4±11.9 nm), which is in agreement with the results obtained for pure *trans*‐**C_1_** (Figures S11, S45). Interestingly, instead of a longitudinal growth, further cooling to 273 K causes a transformation of the short rods into 2D lamellae with similar height and length (*l*_ave_=69.5±15.6 nm) but slightly larger widths (between 20 and 60 nm), which is a product of bundling of the rods already observed at higher temperature (Figure 3 d and Figure S44). This behavior can be rationalized by the simulations, since the alkyl chains surrounding the aromatic core of the stacks potentially offer lateral van der Waals binding sites. This effect, together with the restriction in the degrees of freedom of the stacks containing the distorted *cis* isomer compared to the fibers formed by the pure *trans* isomer, is expected to facilitate the bundling of the rods.

**Figure 3:**
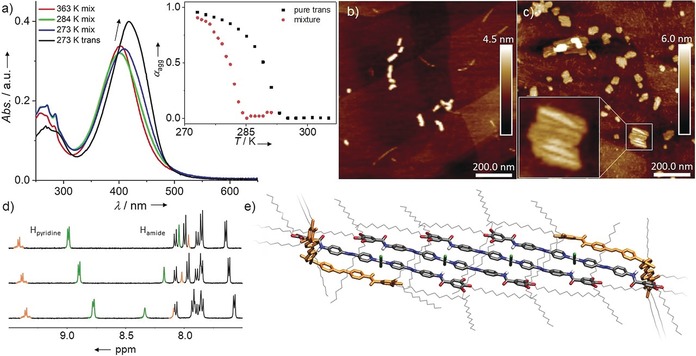
a) VT UV/Vis spectra of a mixture of the two isomers in MCH at 5×10^−5^ m compared with the spectrum of pure **C_1_** at 273 K. Inset: plot of *α_agg_* versus *T* for *trans*‐**C_1_** and a mixture of the two isomers, derived from measuring the spectral changes at 450 nm. b, c) AFM height images of a mixture of both isomers at 2×10^−5^ m in MCH on HOPG spin‐coated at 283 K (b) and 273 K (c). d) VT ^1^H NMR spectra of a mixture of the two isomers (*c*=2.5×10^−3^ m in CDCl_3_) at 323 K (top), 303 K (middle), and 283 K (bottom). e) Dispersion‐corrected PM6 optimized hexameric stack of a 2:1 mixture of *trans* and *cis* isomers.

Ultimately, we validated the reversibility of the system through full recovery of the *trans* form. To this end, the lamellar aggregates from the *cis*+*trans* mixture were heated to the monomer state (363 K) and subsequently irradiated with UV light in order to back‐isomerize the *cis* isomers present (ca. 33 %) to the *trans* form. Cooling the resultant hot solution led to the same aggregation pathway as the freshly prepared *trans*‐**C_1_** in MCH (*c*=2×10^−4^ m, Figure S47). This indicates a nearly quantitative recovery of the *trans* form, as demonstrated by the observation of short fibers by AFM imaging (Figure S48).

In conclusion, we have described a new Pt^II^ complex (**C_1_**) that undergoes both geometrical isomerism and supramolecular polymerization under controlled experimental conditions. While nonpolar media (MCH) induce the formation of thin 1D fibers of pure *trans*‐**C_1_**, the use of more polar solvents (CHCl_3_) to prepare the aggregate solution enables the formation of the distorted *cis* form and leads to attenuated growth into small 2D lamellae. Current work in our lab aims at optimizing the efficiency of coordination isomerism with the ultimate goal of controlling the size of supramolecular assemblies.

## Conflict of interest

The authors declare no conflict of interest.

## Supporting information

As a service to our authors and readers, this journal provides supporting information supplied by the authors. Such materials are peer reviewed and may be re‐organized for online delivery, but are not copy‐edited or typeset. Technical support issues arising from supporting information (other than missing files) should be addressed to the authors.

Supplementary
