# Supplementary material for: Exploiting Coordination Isomerism for Controlled Self‐Assembly
Source: Angew Chem Int Ed Engl. 2019 Sep 9;58(44):15626–30. doi: 10.1002/anie.201908002 (PMC6856968; doi:10.1002/anie.201908002)
Supplement: Supplementary file 1 — Supplementary [file ANIE-58-15626-s001.pdf]

Angewandte Chemie
Eine Zeitschrift der Gesellschaft Deutscher Chemiker

Supporting Information

Exploiting Coordination Isomerism for Controlled Self-Assembly

*Nils Bäumer, Kalathil K. Kartha, Naveen Kumar Allampally, Shiki Yagai, Rodrigo Q. Albuquerque, and Gustavo Fernández**

anie_201908002_sm_miscellaneous_information.pdf

Contents

1. Experimental 3

2. Description of Experimental Techniques.. 6

3. Experimental results for pure *trans* $\mathbf{C_1}$ 7

4. Experimental and theoretical results on the dormancy of azobenzene towards light irradiation in $\mathbf{C_1}$ 16

5. Experimental results for mixtures of *cis* and *trans* $\mathbf{C_1}$ 21

6. Literature 33

1. Experimental

Ligand $\mathbf{L_1}$ and $\mathbf{L_2}$ were synthesized according to previous literature reports.[1] Complexes $\mathbf{C_1}$ and $\mathbf{C_2}$ were synthesized by reaction of the corresponding ligands with $Pt(PhCN)_2Cl_2$ in benzene under reflux.

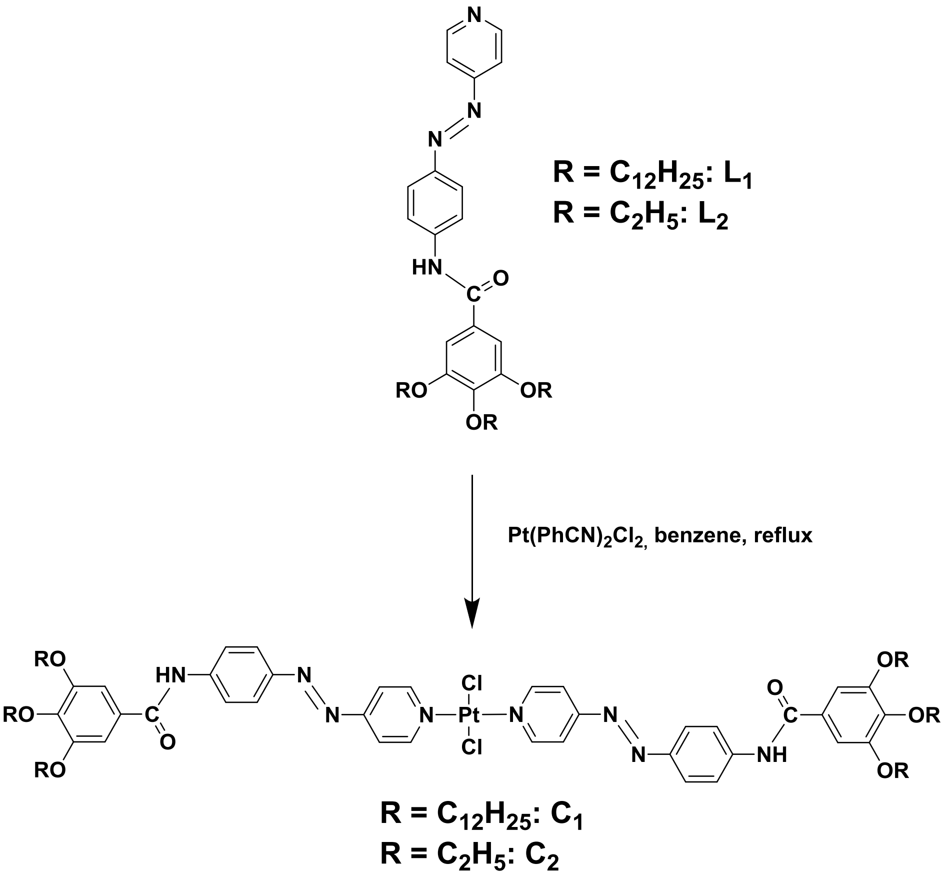

Scheme 1: Synthesis of complexes $\mathbf{C_1}$ and $\mathbf{C_2}$.

Synthesis of $\mathbf{C_1}$: Ligand $\mathbf{L_1}$ (85 mg, 0.10 mmol, 2 eq.) and $Pt(PhCN)_2Cl_2$ (23.6 mg, 0.05 mmol, 1 eq.) were added to benzene (20 mL). The reaction mixture was stirred while refluxing under N_2. The formation of the complex $\mathbf{C_1}$ was regularly followed by ^{1}H-NMR measurements. After 36 hours, $\mathbf{L_1}$ was completely consumed. The reaction mixture was cooled down to room temperature followed by removal of the solvent under reduced pressure. The crude product was purified by several cycles of recrystallization from a mixture of MeOH and Diethyl ether. Yield: 78% ^{1}H-NMR (400 MHz, CD_2Cl_2, 298 K): δ (in ppm) = 8.736 (d, J = 6.1 Hz, 4H, H_a), 8.527 (s, 2H, H_e), 8.045-7.731 (m, 8 H, $H_{c,\,d}$), 7.533 (d, J = 6.3 Hz, 4H, H_b), 7.172 (s, 4H, H_f), 4.157-3.877(m, 12H), 1.857-1.648 (m, 12H), 1.510-1.416 (m, 12H), 1.395-1.221 (m, 96H), 0.997-0.768 (m, 18H). ^{13}C NMR (100.6 MHz, CD_2Cl_2, 298 K): δ(in ppm) = 166.2, 157.8, 155.0, 153.6, 148.6, 144.6, 142.1, 129.7, 125.6, 120.1, 118.3, 106.6, 73.9, 69.7, 32.4, 30.8, 30.0, 26.6, 23.2, 14.4. CHN Analysis: Calculated ($C_{108}H_{172}Cl_2N_8O_8Pt$+$2CH_2Cl_2$), C, 61.55%; H, 8.27%; N, 5.22%;; found C, 61.30%; H, 8.11 %; N,5.07%. FTIR (neat, cm^{-1}): 3275, 2920, 2850, 1660, 1585, 1519, 1501, 1467, 1445, 1420, 1386, 1335, 1303, 1240, 1212, 1143, 1117, 1015, 928, 885, 854, 757, 718, 684, 656, 632, 567, 520.

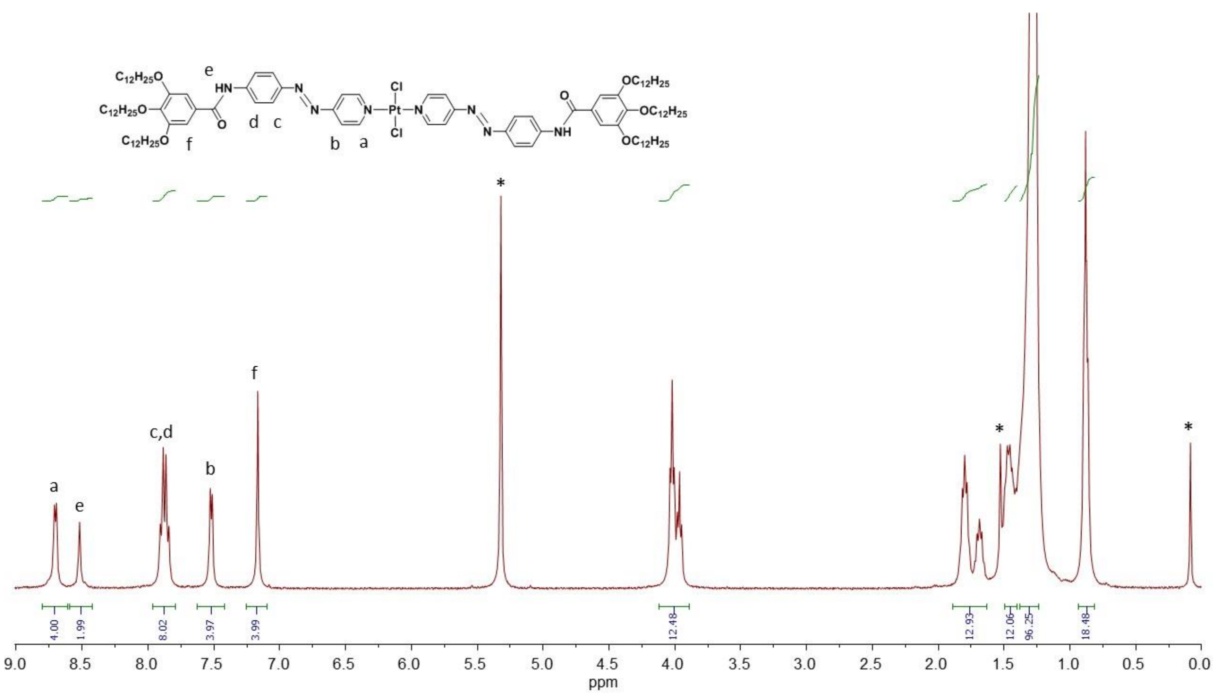

Figure S1: ^{1}H NMR spectrum of **C_1** (400 MHz, CD_2Cl_2, 298 K).

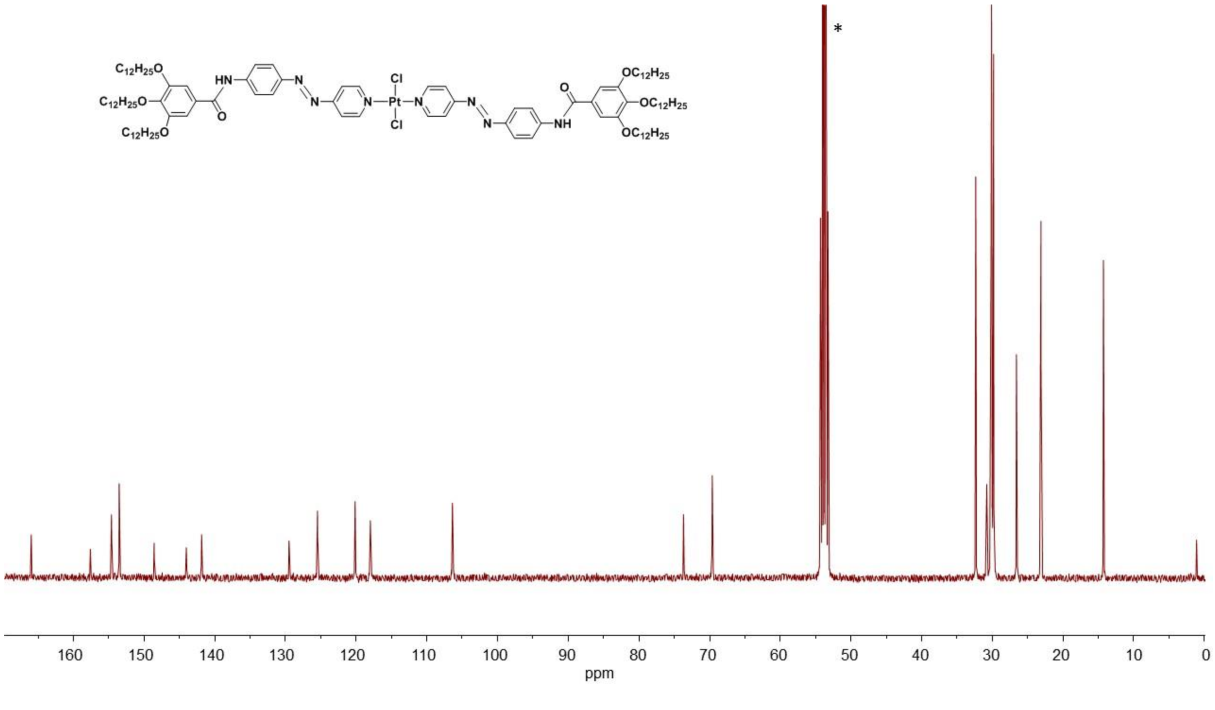

Figure S2: ^{13}C NMR spectrum of **C_1** (100.6 MHz, CD_2Cl_2, 298 K).

Synthesis of C_2: Compound **L_2** (43.3 mg, 2 eq.) and $Pt(PhCN)_2Cl_2$ (23.6 mg, 1 eq.) were added to 20 mL of benzene. The reaction mixture was stirred while refluxing under N_2. The formation of the complex **C_2** was regularly followed by ^{1}H-NMR measurements. After 36 hours, **L_2** was completely consumed. The reaction mixture was cooled down to room temperature followed by removal of the solvent under reduced pressure. The crude product was purified by several cycles of recrystallization from a mixture of EtOAc and MeOH. Yield: 80% ^{1}H-NMR (400 MHz, DMSO-d_6, 298 K): δ (in ppm) = 10.547 (s, 2H, H_e), 9.138-8.757 (m, 4H, H_a), 8.071(s, 8H, $H_{c,d}$), 7.853 (d, J = 7.2 Hz, 4H, H_b), 7.294 (s, 4H, H_f), 4.1834.091(m, 8H), 4.076-3.998(m,

4H), 1.414-1.349(m, 12H), 1.297-1.229 (m, 6H). ^{13}C NMR (100.6 MHz, DMSO-d_6, 298 K): δ (in ppm) = 165.6, 157.6, 155.4, 152.3, 147.9, 144.8, 140.5, 125.2, 120.7, 17.8, 106.7, 69.0, 64.4, 15.6, 14.6. FTIR (neat, cm^{-1}): 3316, 3102, 2976, 2933, 2896, 1656, 1584, 1525, 1498, 1425, 1397, 1329, 1300, 1239, 1202, 1029, 901, 849, 750, 658, 632, 561, 522.

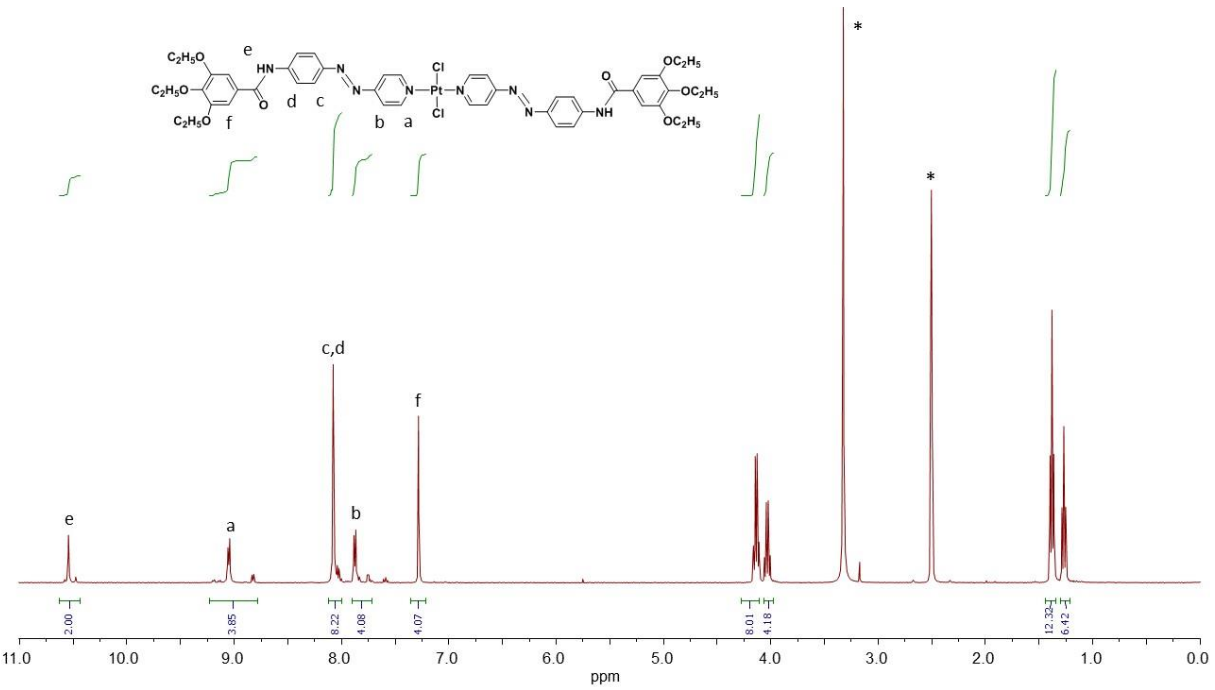

Figure S3: ^{1}H NMR spectrum of **C2** (400 MHz, DMSO-d_6, 298 K).

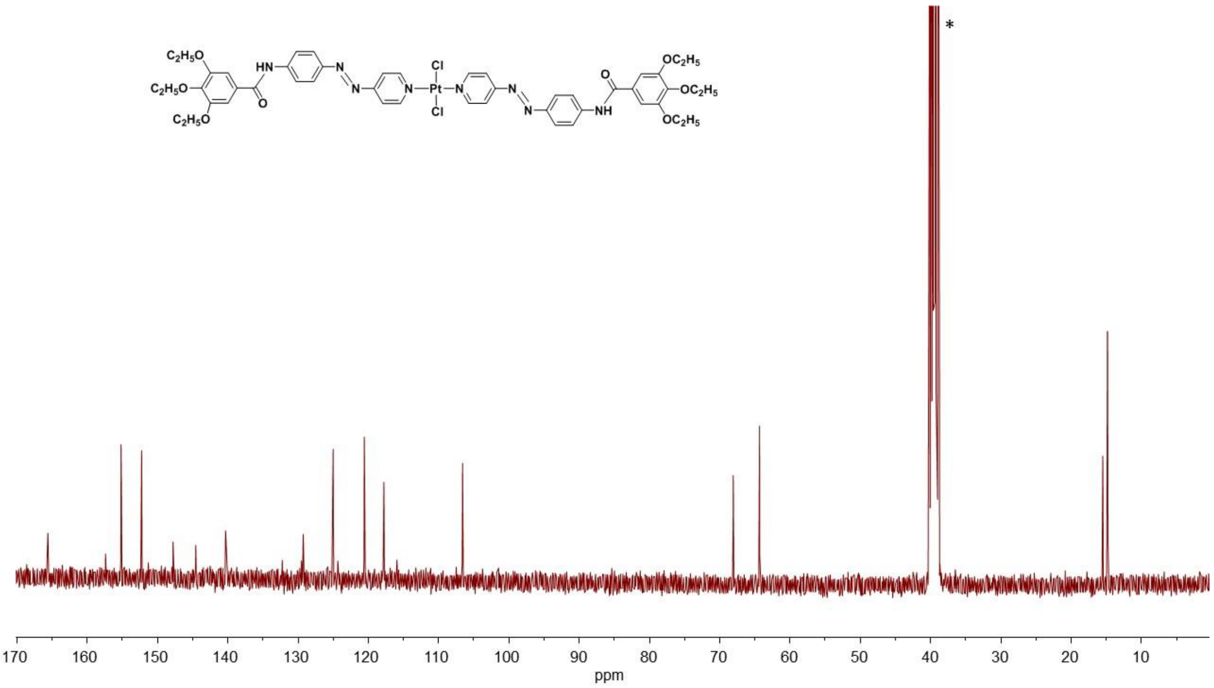

Figure S4: ^{13}C NMR spectrum of **C2** (100.6 MHz, DMSO-d_6, 298 K).

2. Description of Experimental Techniques

General Procedures: All solvents were dried according to standard procedures. Reagents were used as purchased. All air-sensitive reactions were carried out under argon or nitrogen atmosphere.

NMR-measurements: ^{1}H and ^{13}C NMR spectra were recorded on a *Bruker Avance 400* (^{1}H: 400 MHz; ^{13}C: 100.6 MHz) and a *Bruker AV300* (^{1}H: 300 MHz; ^{13}C: 100.6 MHz). Additional 1D ^{1}H as well as 2D H,H-COSY and 2D H,H ROESY spectra were recorded on an *Agilent DD2 500* (^{1}H: 500 MHz) and an *Agilent DD2 600* (^{1}H: 600 MHz) at a standard temperature of 298 K in deuterated solvents. Deviating temperature is explicitly mentioned when used. The recorded spectra were referenced to the remaining resonance signals of the deuterated solvents ($CDCl_3$: 7.26 ppm (^{1}H); DCM: 5.32 ppm (^{1}H)). The coupling constant *J* of the measured spin multiplets is given in Hertz (Hz) and the chemical shifts are given in reference to the chemical shift of trimethylsilane (0 ppm). The abbreviations used to analyze the recorded spectra are: s (singlet), d (doublet), m (multiplet).

Mass spectroscopy: MALDI-mass spectra were recorded on an *Autoflex Speed* manufactured by *Bruker Daltronics*. A *SmartBeam^{TM}* NdYAF-Laser with a wavelength of 335 nm was used. The signals are described by their mass/charge ratio (m/z) in u.

Elemental Analysis: The elemental analysis was carried out using a *vario EL III Element Analyzer* by *elementar Analysensysteme GmbH*.

UV-Vis spectroscopy: All UV-Vis spectra were recorded on a *V-770* and a *V-750* by the company *JASCO* with a spectral bandwidth of 1.0 nm and a scan rate of 400 nm min^{-1}. Glass cuvettes with an optical length of 1 cm and 1 mm were used. All measurements have been conducted in solvents from commercial sources from spectroscopic grade.

Dynamic Light Scattering: All DLS spectra have been recorded on a *CGS-3 Compact Goniometer System* manufactured by *ALV GmbH*, equipped with a HeNe Laser with a wavelength of 632.8 nm (22 mW) and an *ALV/LSE-5004 Digital Correlator* by *ALV GmbH*.

Atomic force microscopy: The AFM images have been recorded on a *Multimode®8 SPM Systems* manufactured by *Bruker AXS*. The used cantilevers were *AC200TS* by *Oxford Instruments* with an average spring constant of 9 N m^{-1}, an average frequency of 150 kHz, an average length of 200 µM, an average width of 40 µM and an average tip radius of 7 nm. All solutions have been spin-coated onto an HOPG surface using a spin rate between 2000 and 4000 rpm.

Irradiation Methods: Irradiation-based experiments were performed using a LED *LED Engin LZ1-10UV00-0000* by *Mouser electronics* at 365 nm. For other wavelengths, LEDs by *Conrad Electronics* were used. *HighPower-LED Grün 87 lm 130° 3.8 V 1000 mA Roschwege LSC-G* for measurements using 520 nm wavelength, H*ighPower-LED Rot 52 lm 130° 3.5 V 1000 mA Roschwege LSC-R* for measurements using 620 nm wavelength and *HighPower-LED Blau 31 lm 130° 2.3 V 700 mA Roschwege LSC-B* for measurements using 465 nm wavelength. Photoirradiation in the photoreactor was performed using either a *Photoreaktor 400m Blende* (Hg light source; λ = 254 nm, max 40 W light intensity) from *Gräntzel Karlsruhe* or using a *Black Ray® B100-Ap High Intensity UV Lamp* (λ = 365 nm, 100 W).

3. Experimental results for pure *trans*-**C1**

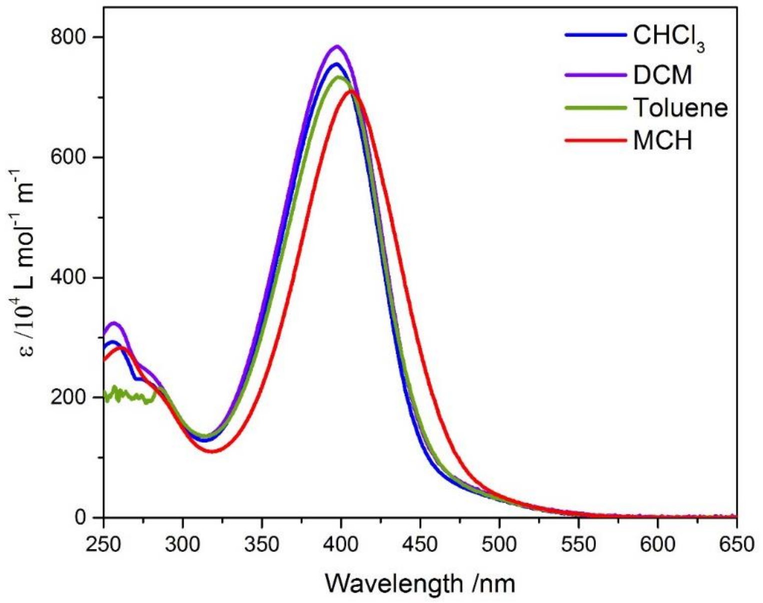

Figure S5: Solvent-dependent UV-Vis studies of **C1** at 1 x 10^{-5} M.

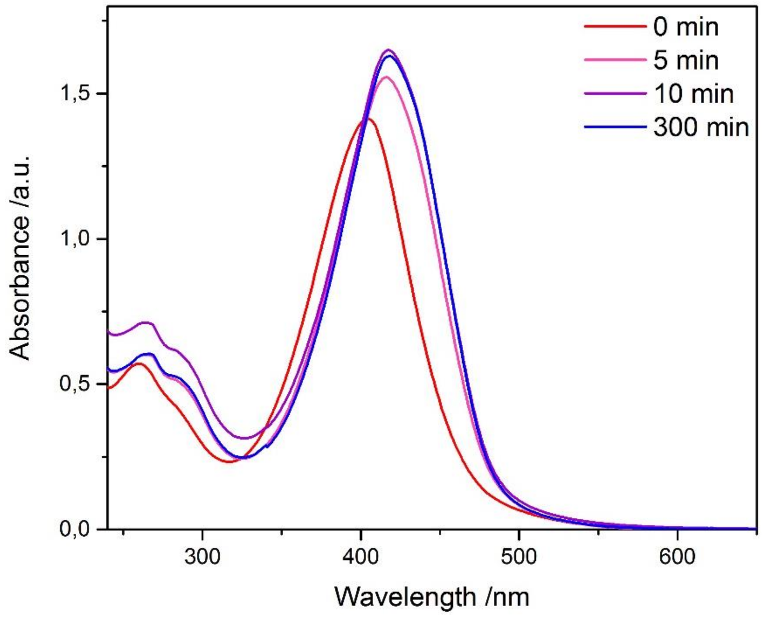

Figure S6: Time-dependent UV-Vis studies of **C1** (MCH, 2 x 10^{-5} M) after rapid cooling (quenching) from 363 to 273 K.

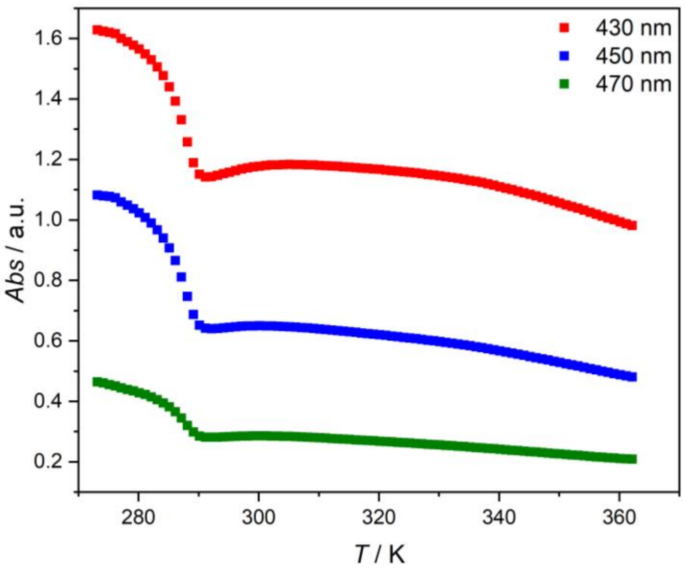

Figure S7: Plots of *Abs vs. T* at different wavelengths of a 2 x 10^{-5} M solution of **C_1** in MCH. The unusual two-step curve can be observed independent of the wavelength monitored.

$$E = \varepsilon \cdot c \cdot d \tag{1}$$

$$\alpha_{agg} = 1 - \frac{\varepsilon(T) - \varepsilon_{agg}}{\varepsilon_{mon} - \varepsilon_{agg}} \tag{2}$$

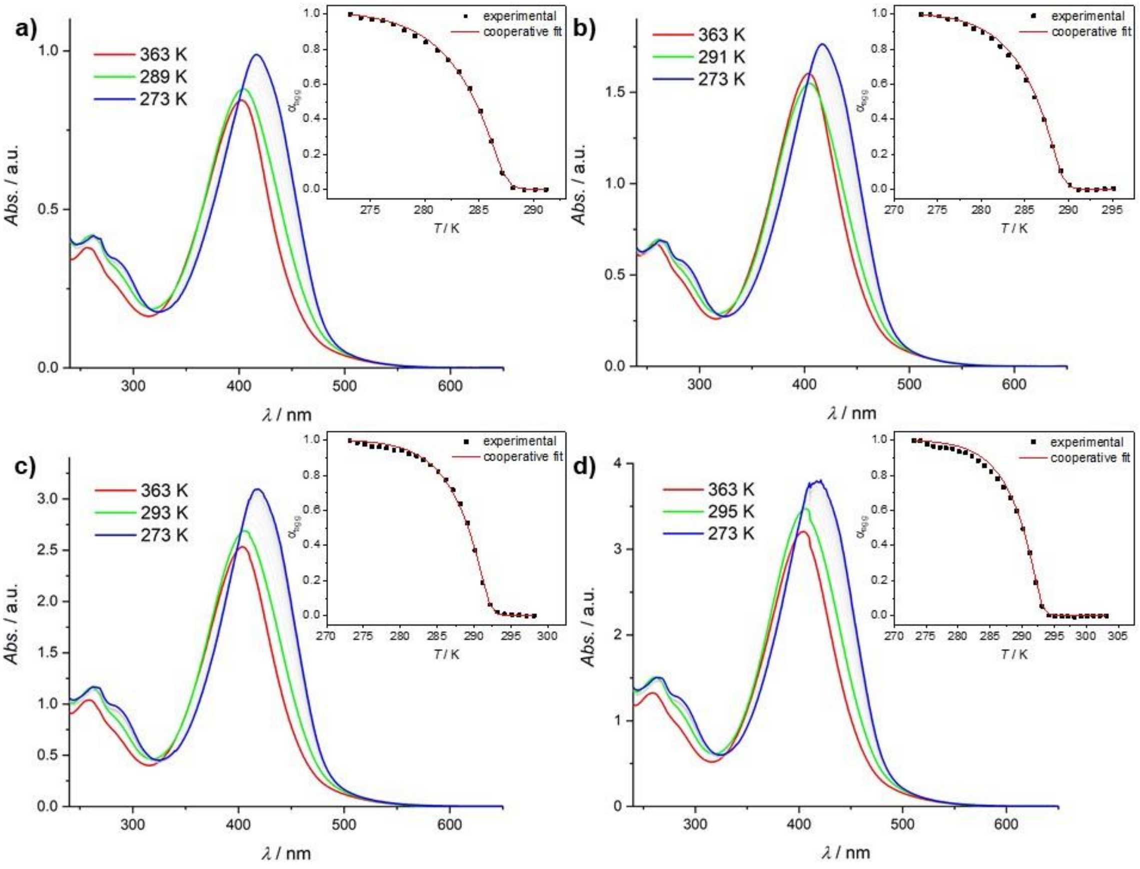

Figure S8: VT UV-Vis experiments of **C_1** at different concentrations (1 x 10^{-5}, 1.6 x 10^{-5}, 3.2 x 10^{-5} and 4 x 10^{-5} M), corresponding cooling curves obtained by monitoring the spectral changes at 450 nm and fits to the nucleation-elongation model.[2] The corresponding plots α_{agg} *vs*. *T* have been obtained by applying equation 1 and 2 to the plots of the absorbance at 450 nm against the temperature.

Table S1: Thermodynamic parameters K_{nucl}, K_{el}, σ, T_{el}, ΔH^0 and ΔH^0_{nucl} derived from the VT UV-Vis experiments of **C1** (MCH, λ = 450 nm) on the basis of the nucleation-elongation model using a global fitting.[2]

Conc. /M	ΔH^0 /kJ mol^{-1}	ΔH^0_{nucl} /kJ mol^{-1}	T_{el} /K	K_{nucl}	K_{el}	σ
1.0 x 10^{-5}	-172.51	-17.02	287.2	7.33	7036.96	1.04 x 10^{-3}
1.6 x 10^{-5}	-172.51	-17.02	289.1	7.33	7036.96	1.04 x 10^{-3}
3.2 x 10^{-5}	-172.51	-17.02	291.9	7.33	7036.96	1.04 x 10^{-3}
4.0 x 10^{-5}	-172.51	-17.02	292.8	7.33	7036.96	1.04 x 10^{-3}

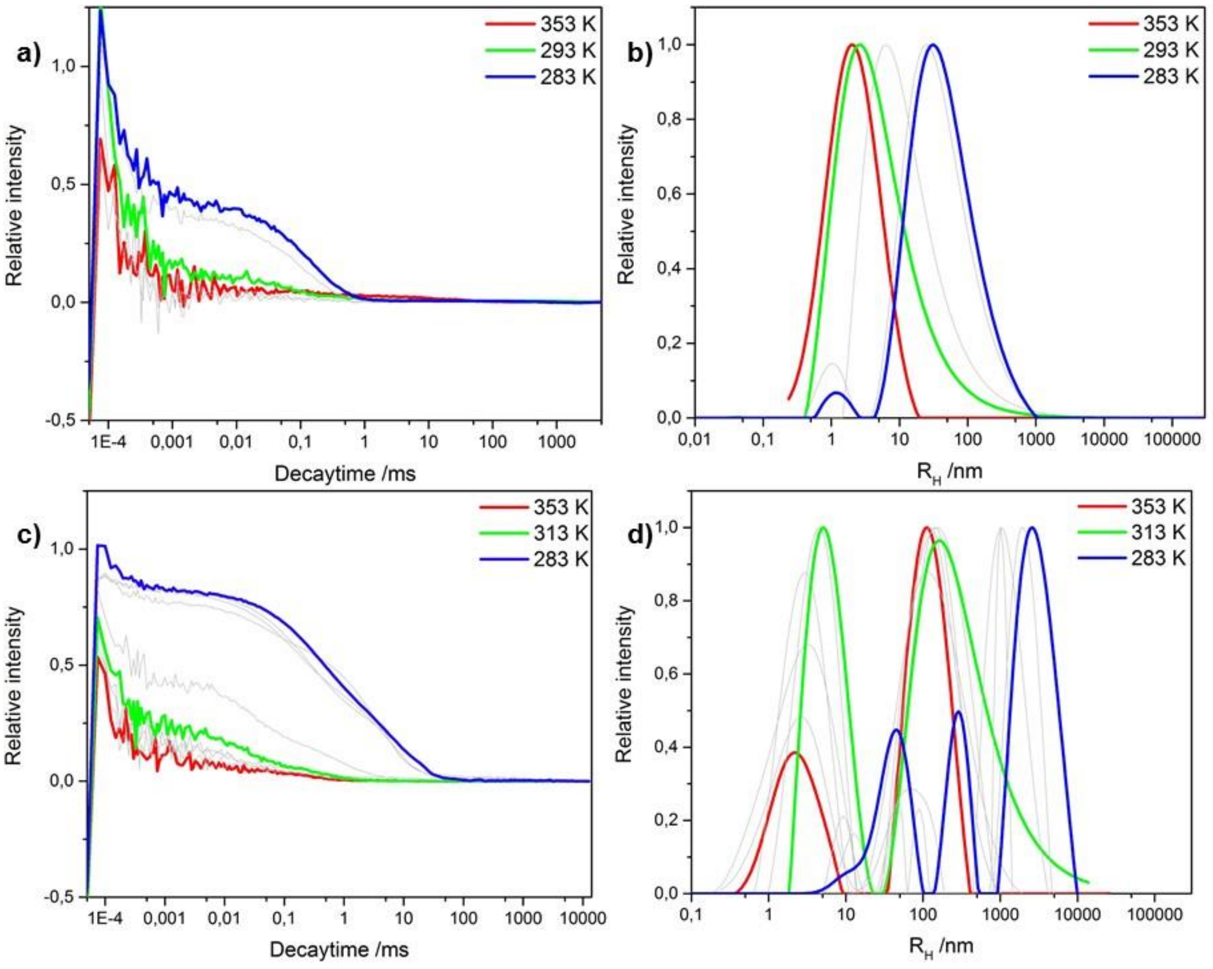

Figure S9: VT DLS studies of **C1** in MCH (a) c = 5 x 10^{-5} M; correlation function; b) c = 5 x 10^{-5} M; distribution function; c) c = 2 x 10^{-4} M; correlation function; d) c = 2 x 10^{-4} M; distribution function.

The very small particle sizes extracted from DLS validate the previous hypothesis that the initial pre-nucleation event is primarily due to molecular conformational changes rather than to an oligomerization process.

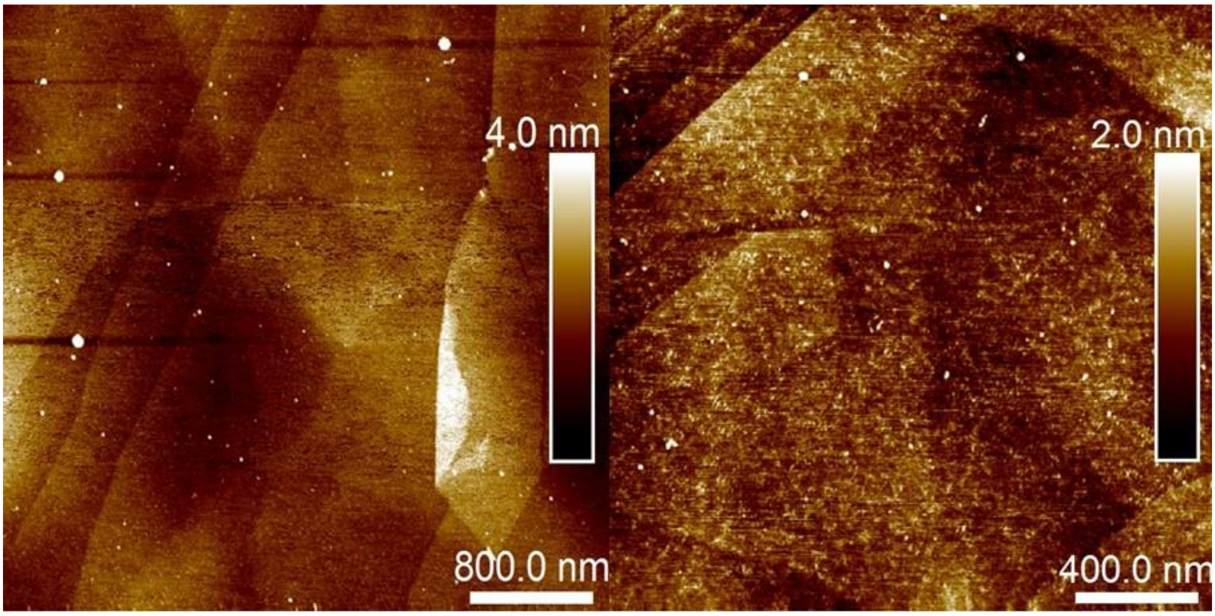

Figure S10: AFM height image of a solution of **C_1** in MCH at 5 x 10^{-5} M after cooling a solution from 363 to 313 K with a cooling rate of 0.2 K/min (spin-coated at 363 K (left) and at 313 K (right)).

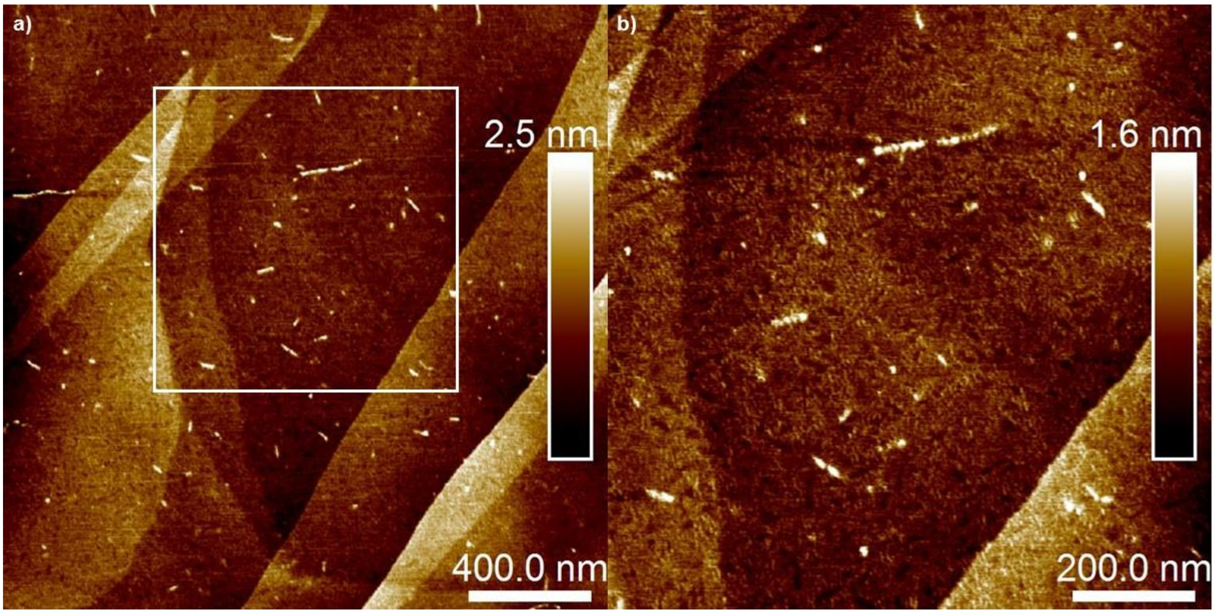

Figure S11: AFM height images of aggregates formed by **C_1** in MCH at 5 x 10^{-5} M after cooling a solution from 363 to 293 K with a cooling rate of 0.2 K/min (a)) and a more zoomed in image (b)).

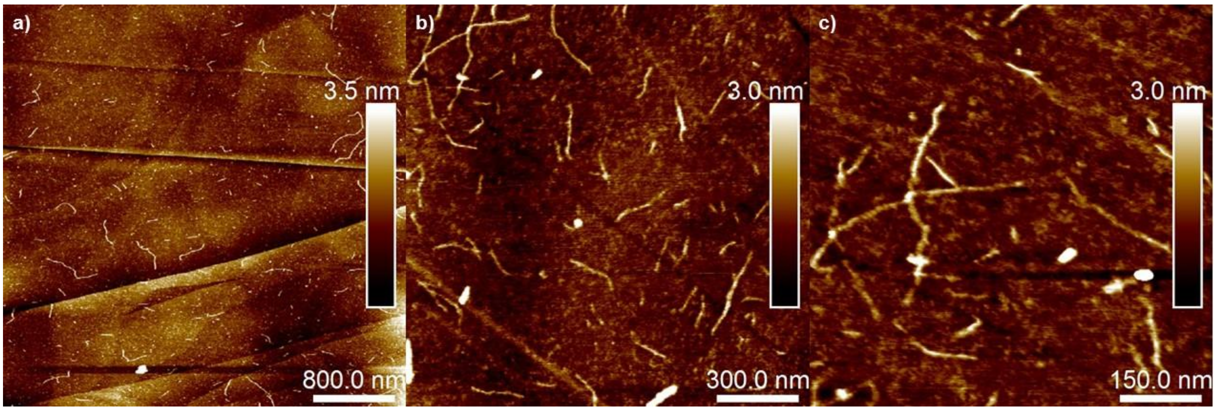

Figure S12: AFM height images of aggregates formed by **C_1** in MCH after cooling a solution from 363 to 273 K with a cooling rate of 0.2 K/min at different concentrations. a) 5 x 10^{-4} M; b)/c) 1 x 10^{-3} M.

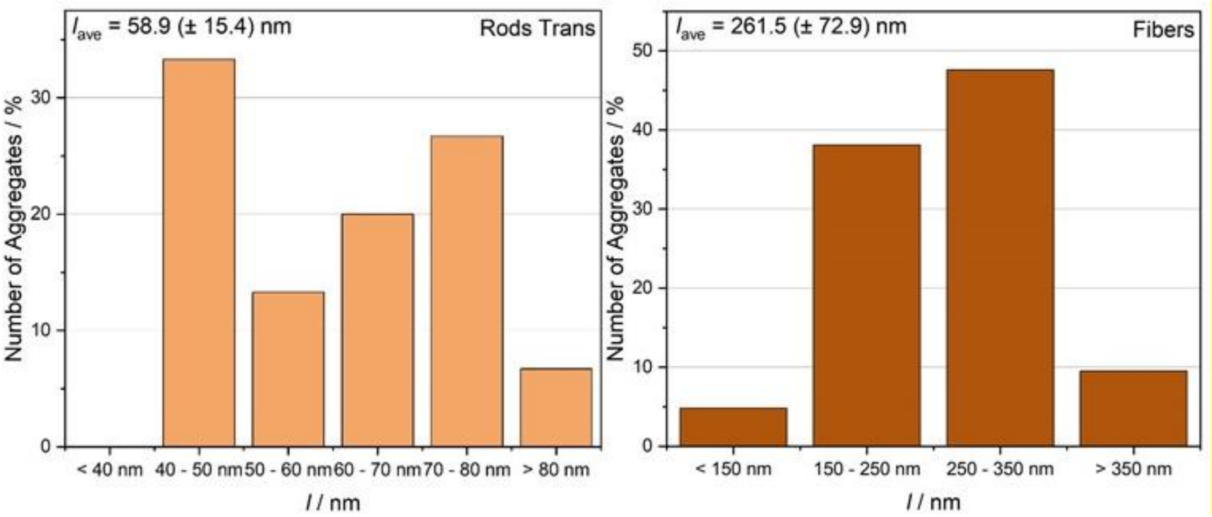

Figure S13: Size distribution of the polymer lengths as calculated from AFM images of the aggregates formed by **C_1** in MCH after cooling a solution from 363 to 293 (left) and 273 K (right) with a cooling rate of 0.2 K/min at 5 x 10-5 M.

The signals corresponding to protons H_e, H_d and H_f show a downfield shift, indicating the proximity of an electron rich group such as O or Cl and the possible involvement in weak hydrogen bonding. On the other hand, the signals of protons H_a, H_b and H_c exhibit an upfield shift upon aggregation due to π-π stacking. Furthermore, all signals broaden under the applied conditions.

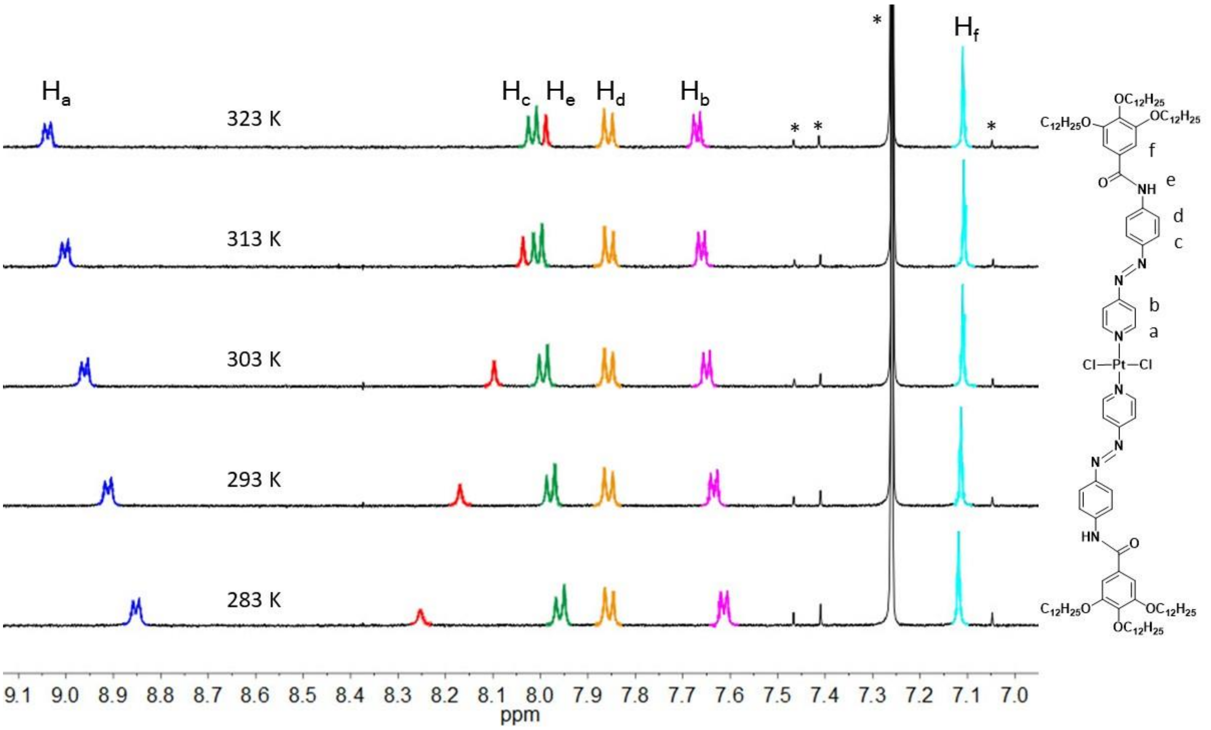

Figure S14: Temperature-dependent ^{1}H NMR spectra of **C_1** at 1 x 10^{-3} M between 323 and 283 K (600 MHz, $CDCl_3$).

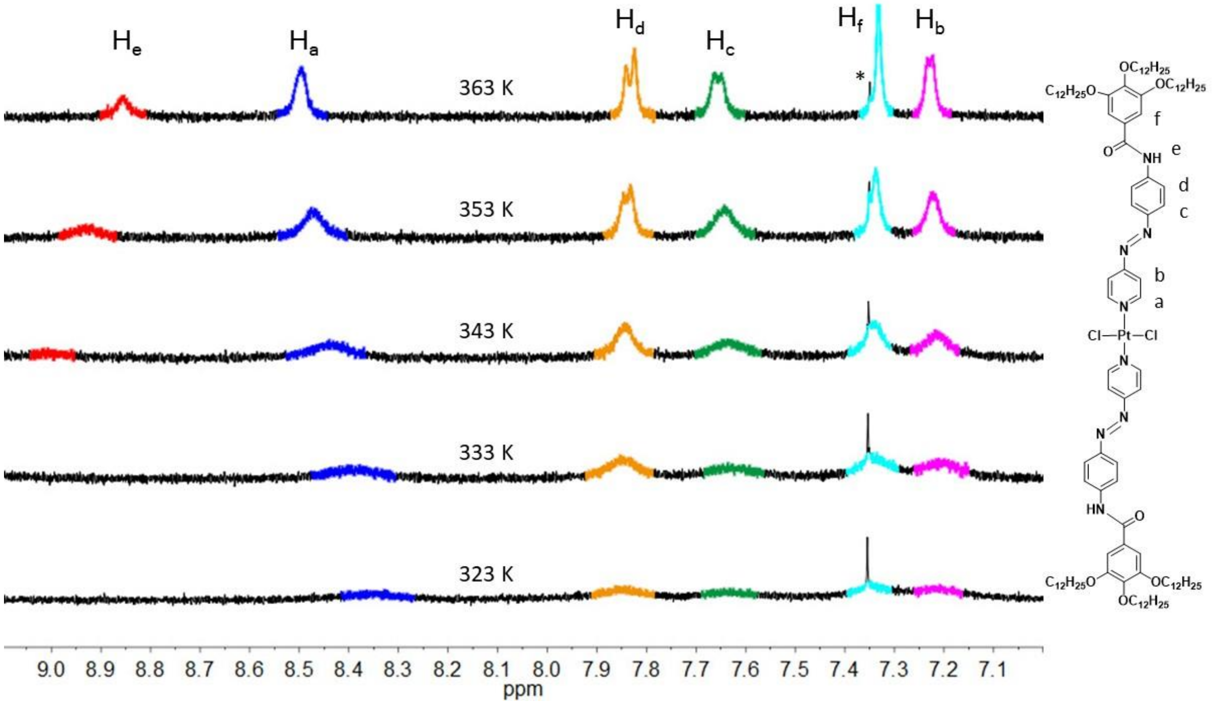

Figure S15: Temperature-dependent ^{1}H NMR spectra of **C_1** at 1 x 10^{-3} M between 363 and 323 K (600 MHz, MCH-d_{14}).

COSY- and ROESY-^{1}H-NMR studies provided further insight into the packing mode of the **C$_1$** aggregates. Correlation signals between proton H_a and protons H_f and H_d as well as an additional cross signal originating from the interaction between protons H_c and H_b are apparent, leading to the plausible slipped stack packing driven by N-H···Cl interactions. In addition, the signals corresponding to the intermolecular interactions of the protons H_b and H_a and the protons corresponding to the first methylene unit of the alkoxy side chain were also observed underlining the proposed arrangement of **C$_1$** in the aggregated state.

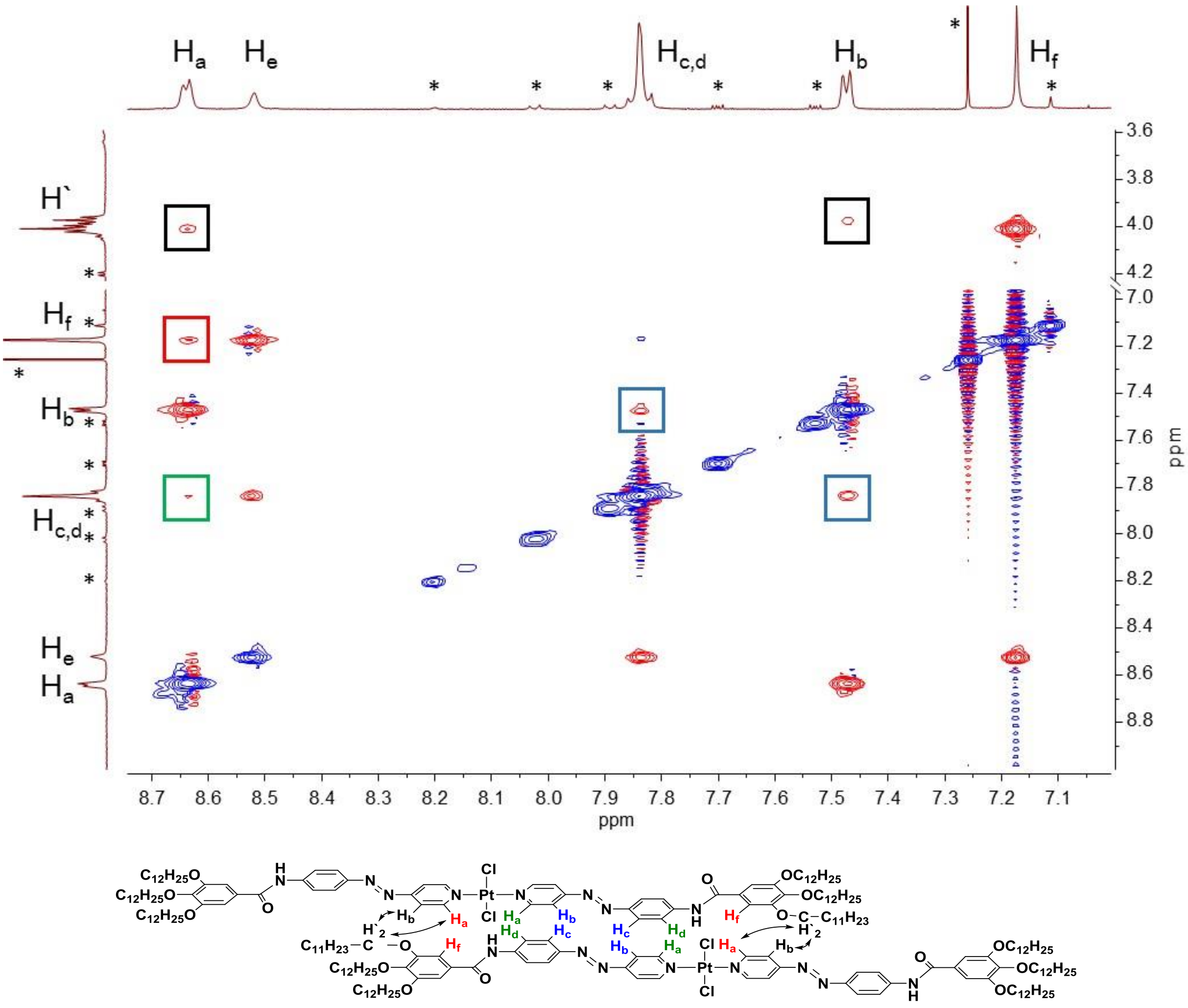

Figure S16: Overlay of COSY and ROESY spectra of **C$_1$** at 1 x 10^{-2} M (600 MHz, $CDCl_3$, 298 K). Boxes indicate the signals observed only by ROESY NMR (intermolecular). The observed coupling pattern (protons H_a and H_f (red box), H_a and $H_{c,d}$ (green box), H_b and $H_{c,d}$ (blue box) and H_a/H_b with H` (black boxes)) is in agreement with the VT NMR and VT UV-Vis results and support the stacking in a slipped fashion with the possible spatial arrangement depicted at the bottom of the Figure.

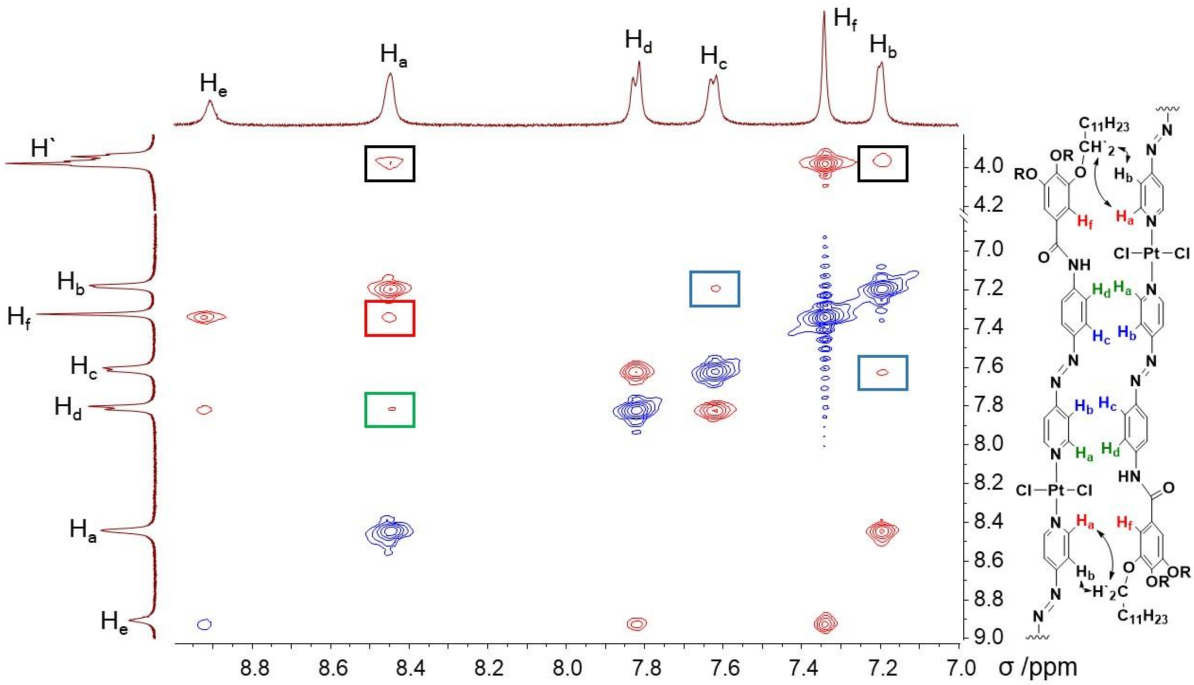

Figure S17: Overlay of COSY and ROESY spectra of **C1** at 7.5 x 10^{-3} M (600 MHz, MCH-d_{14}, 363 K). Boxes indicate the signals observed only by ROESY NMR (intermolecular). The observed coupling pattern (protons H_a and H_f (red box), H_a and $H_{c,d}$ (green box), H_b and $H_{c,d}$ (blue box) and H_a/H_b with H` (black boxes)) is in agreement with the VT NMR and VT UV-Vis results and support the stacking in a slipped fashion with the possible spatial arrangement depicted on the right of the image.

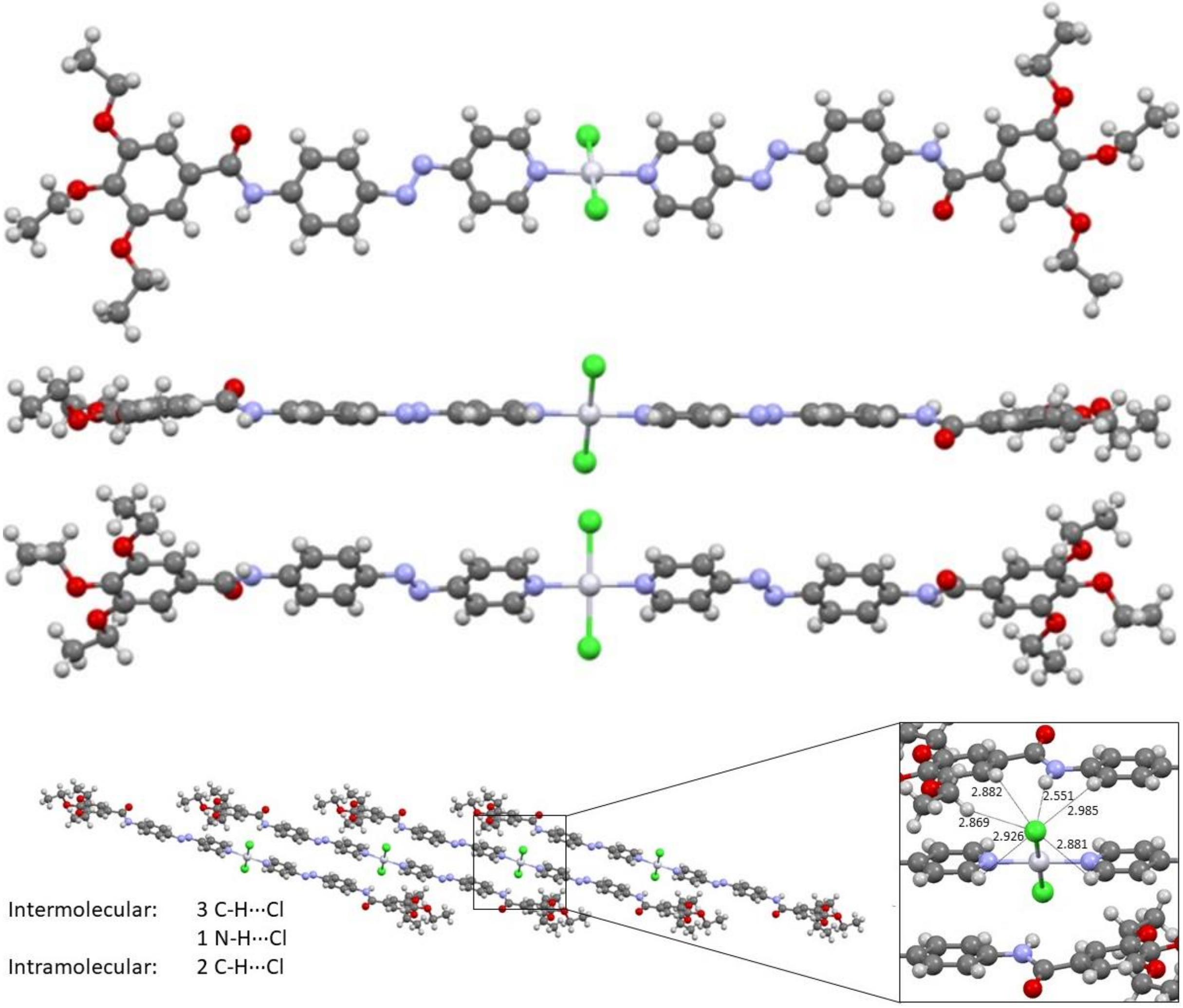

Figure S18: Crystal structure analysis of **C₂** in three different views (front, side and diagonal) with the molecular packing and the most important inter- and intramolecular interactions depicted above (distances given in Å).

4. Experimental and theoretical results on the dormancy of azobenzene towards light irradiation in **C₁** (upon Pt^{II} coordination)

Density Functional Theory (DFT) and Time-Dependent DFT calculations at the PBE0/LANL2DZ/6-31G* level predict different excitation processes with strong MLCT character (Fig. S19 and S20). Once populated, these excited states can be non-radiatively depopulated very efficiently, whose kinetics have been reported to suppress photoisomerization of azobenzene groups inside the same complex.[3] In very good accordance with the theoretical predictions, irradiation of the fibers of **C_1** with 365 nm UV light causes negligible absorption changes, even after prolonged exposure time or using other irradiation wavelengths (Figs. S21, 22). The insensitivity of **C_1** aggregates to light was additionally demonstrated by the absence of new resonances and signal shifts in the ^{1}H NMR spectra of **C_1** aggregates (c = 1 x 10^{-3} M) in CDCl3/MCH-d_{14} (1:9) upon irradiation (Fig. S23, 24). Furthermore, AFM images taken after different irradiation periods (Fig. S25) demonstrate no effect of light on the nanoscale morphology, except a negligible reduction in fiber length that is ascribed to internal heat created by the excitation process.

Excited State 1: T1 1.6726 eV 741.26 nm f=0.0000 <S**2>=2.000

486 -> 497	-0.28564
486 -> 498	-0.31226
**487 -> 497**	**0.38749**
487 -> 498	0.35306

250 pts (MOs gabedit), 95 % isosurface

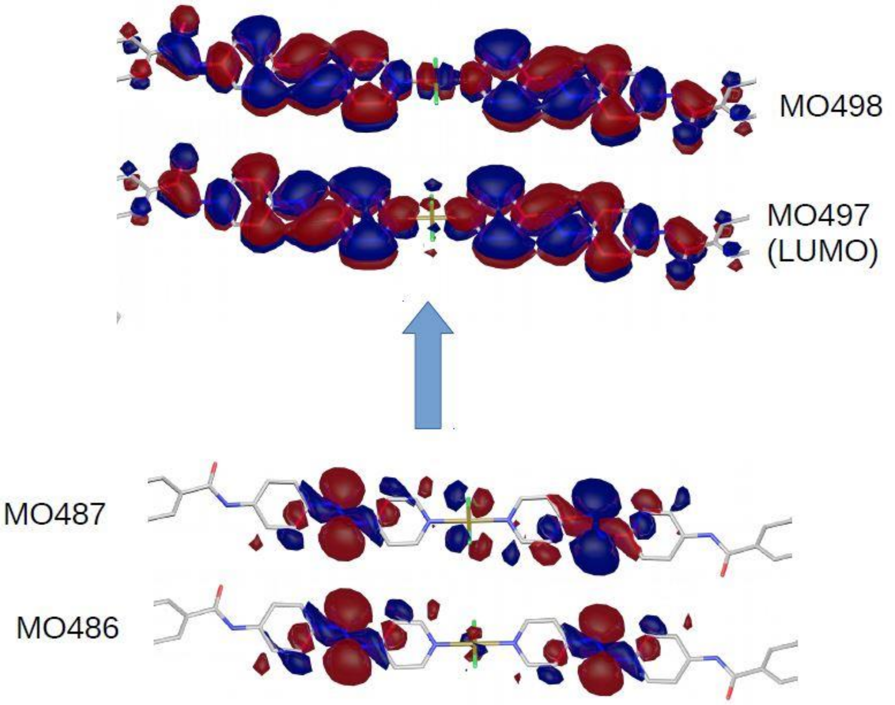

Figure S19: Calculated molecular orbitals for the lowest triplet state (= emitting state) with clear LC and LMCT character.

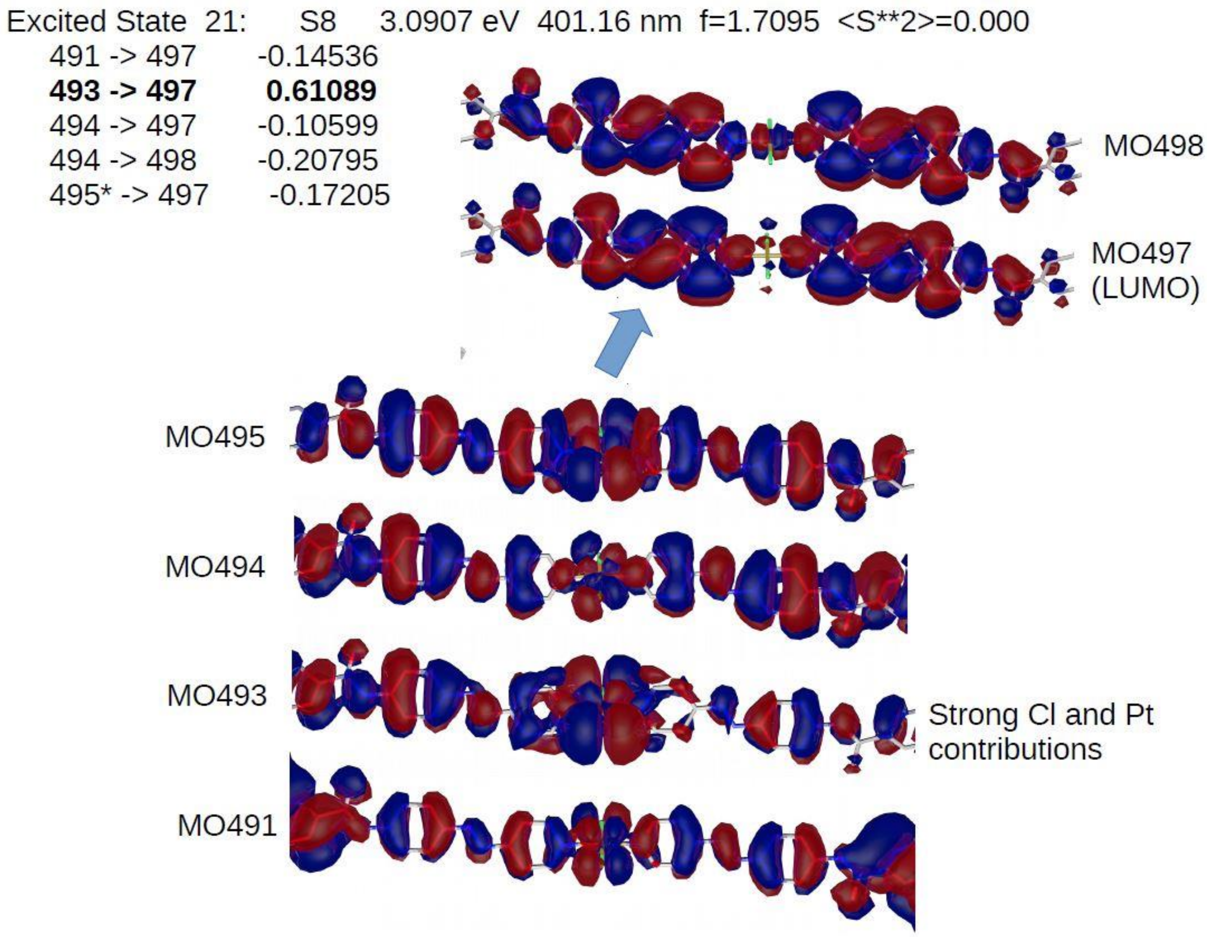

Figure S20: Calculated molecular orbitals for the excited S8 state (= maximum absorbance) with clear MLCT character.

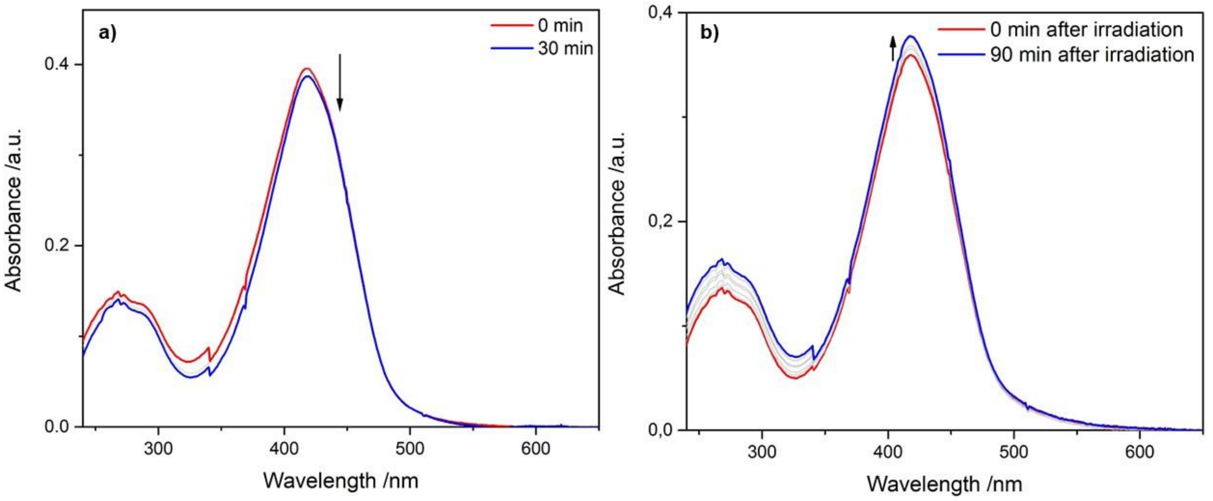

Figure S21: a) Time-dependent UV-Vis spectra of $\mathbf{C_1}$ in MCH (c = 5 x 10^{-5} M, 273 K, l = 1 mm) during irradiation with a black arrow indicating the spectral changes. b) time-dependent UV-Vis spectra after the irradiation process was terminated with an arrow indicating the spectral changes.

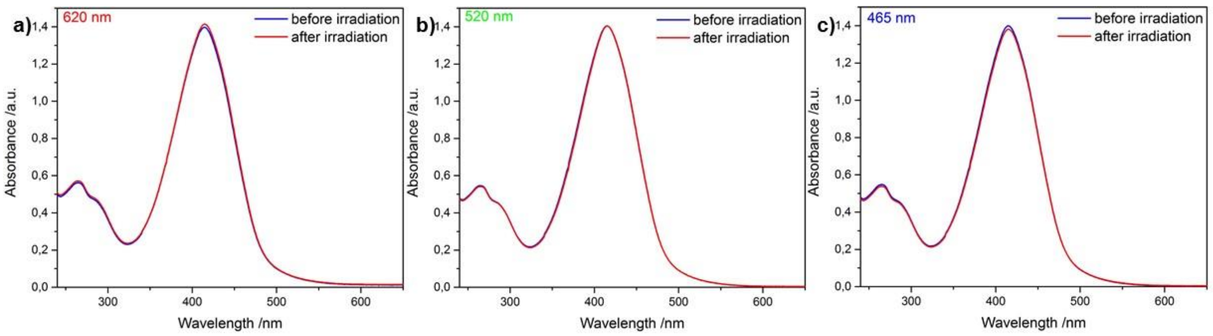

Figure S22: UV-Vis spectra of $\mathbf{C_1}$ in MCH (c = 2 x 10^{-5} M, 273 K, l = 1 cm) before and after irradiation for 30 min. with a LED emitting at a) 620 nm, b) 520 nm) and c) 465 nm.

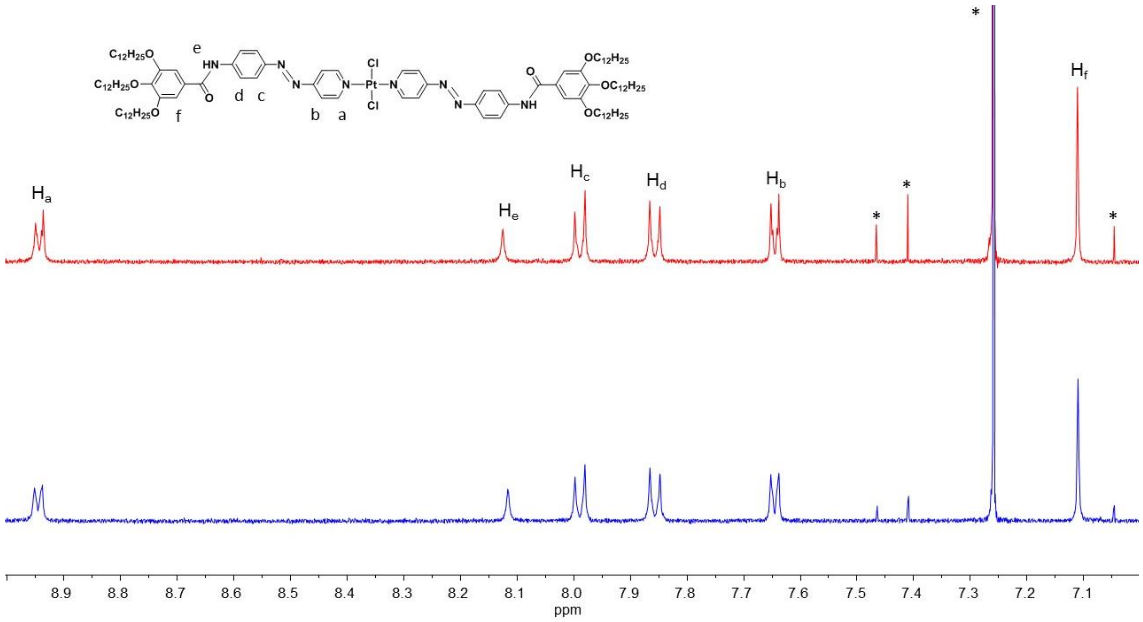

Figure S23: ^{1}H NMR spectra of **C_1** at 1 x 10^{-3} M (600 MHz, $CDCl_3$, 298 K) before and after irradiation for 15 min. in a photoreactor at 365 nm.

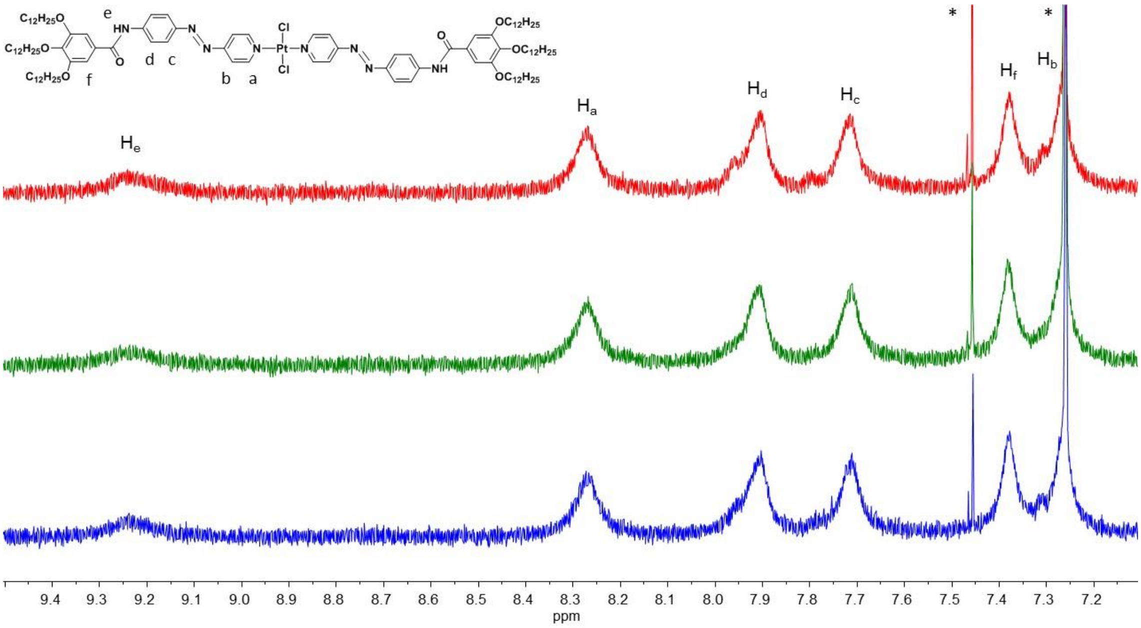

Figure S24: ^{1}H NMR spectra of **C_1** at 1 x 10^{-3} M (600 MHz, CDCl3/MCH-d_{14} (1:9), 298 K) before (top) and after irradiation for 15 min. in a photoreactor at 365 nm (middle) and 250 nm (bottom).

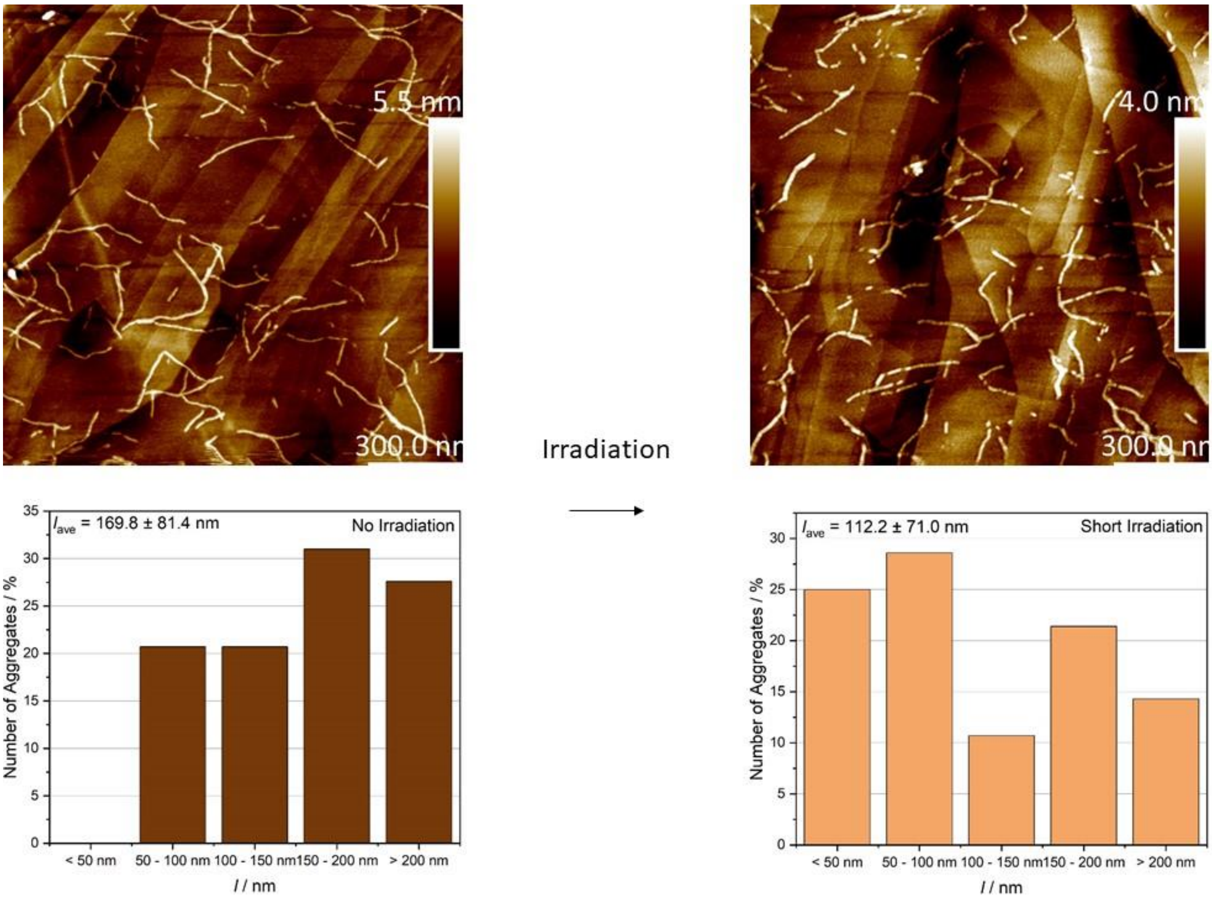

Figure S25: AFM height images of aggregates formed by **C1** in MCH after cooling a solution from 363 to 273 K with a cooling rate of 0.2 K/min at 3 x 10^{-5} M before irradiation (top left) and after 15 min. of irradiation (top right). Length distribution of the fibers as calculated from the respective AFM images (Below). The AFM images confirm the dormant nature of the fibers to UV-irradiation, as only a very minor reduction in the fiber length can be observed, which is ascribed to temperature effects caused by the UV-LED next to the cooled cuvette as well as the formation of internal heat by the excitation process.

5. Experimental results for mixtures of *cis*- and *trans*-**C₁**

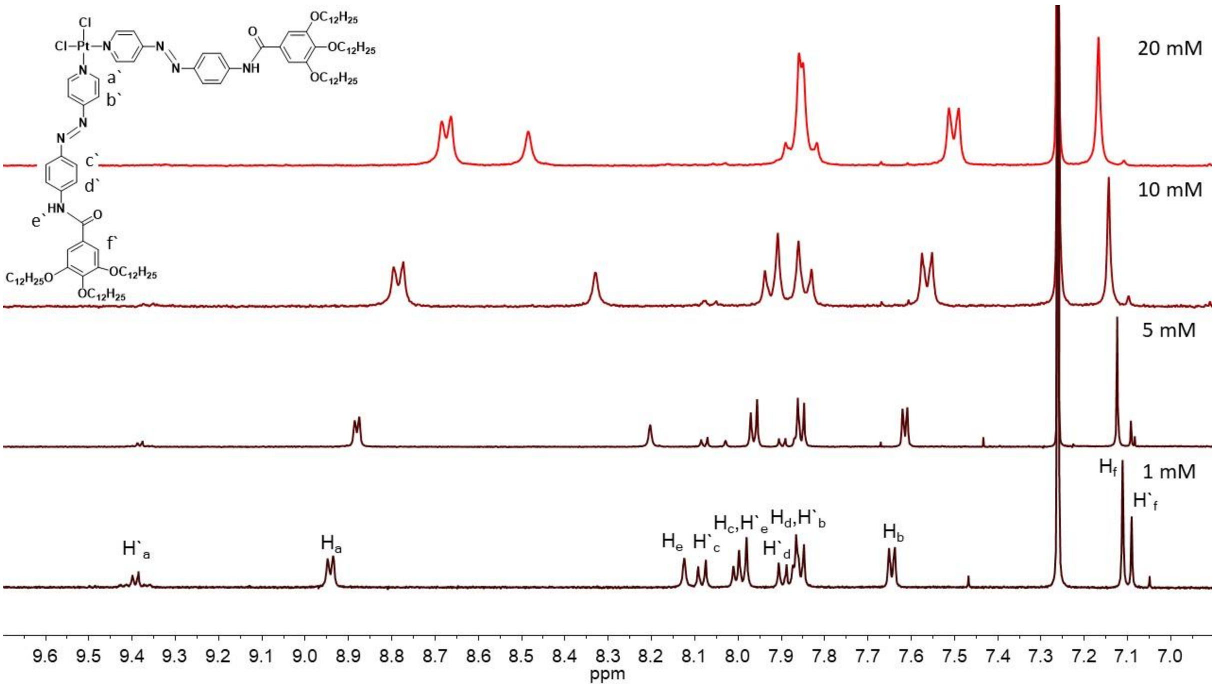

Figure S26: Concentration-dependent ^{1}H NMR spectra of **C_1** between 20 mM and 1 mM showing an increase in the content of the *cis*-isomer upon dilution (20 x 10^{-3} M: 2% (first spectrum), 10 x 10^{-3} M: 5% (second spectrum), 5 x 10^{-3} M: 13% (third spectrum), 1 x 10^{-3} M: 33% (fourth spectrum) (600 MHz, $CDCl_3$, 298 K).

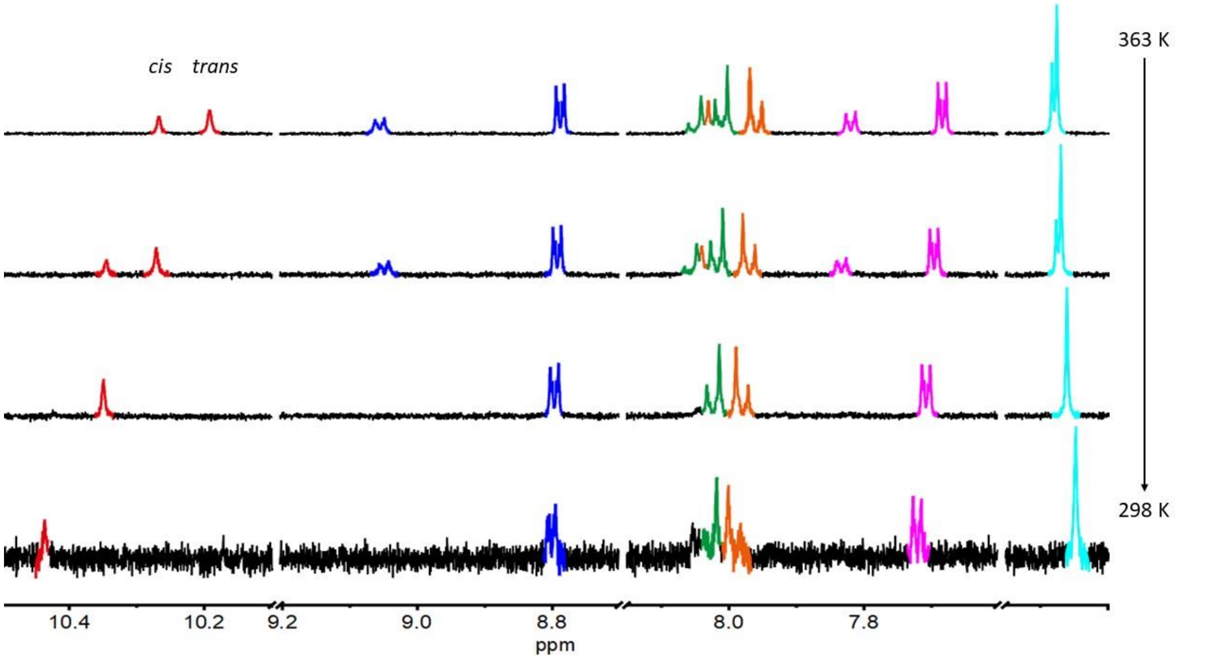

Figure S27: VT ^{1}H NMR studies of a mixture of both isomers of **C_1** at 1 x 10^{-3} M ((600 MHz, DMSO-d_6) between 363 and 298 K. With isomeric ratios of: 40% cis/ 60% trans (363 K); 35% cis/ 65% trans (343 K); 100% trans (323 K); 100% trans (298 K).

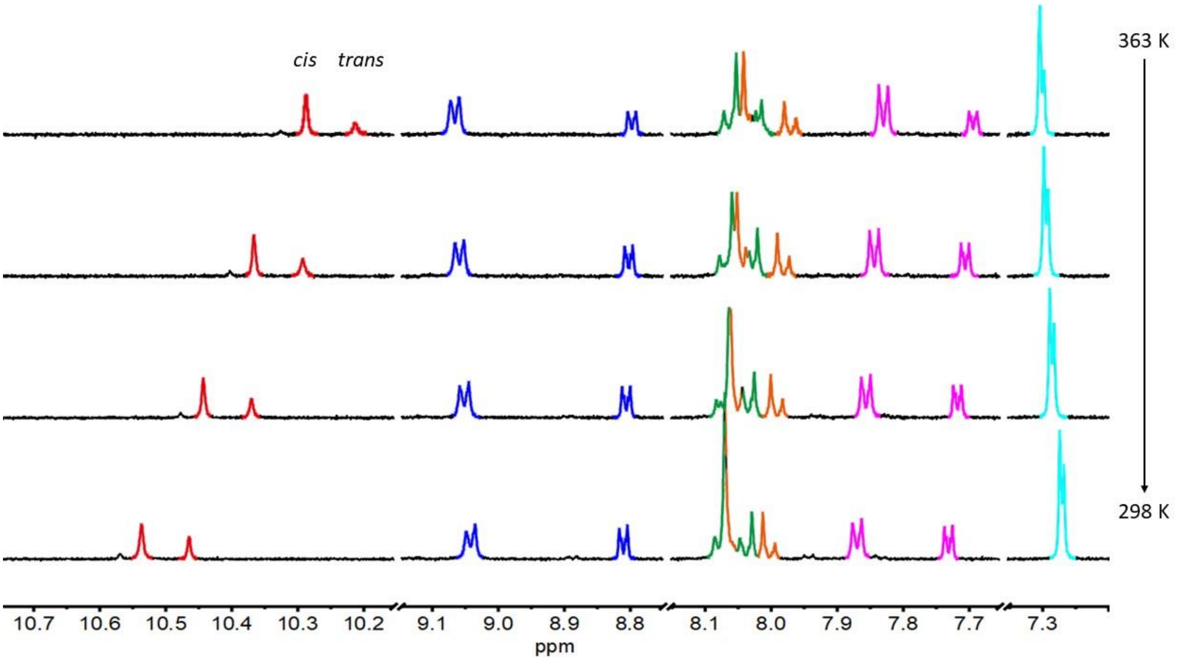

Figure S28: VT ^{1}H NMR studies of a mixture of both isomers of **C_2** at 1 x 10^{-3} M ((600 MHz, DMSO-d_6) between 363 and 298 K. With isomeric ratios of: 73% cis/ 27% trans (363 K); 67% cis/ 33% trans (343 K); 66% cis/ 34% trans (323 K); 65% cis/ 35% trans (298 K).

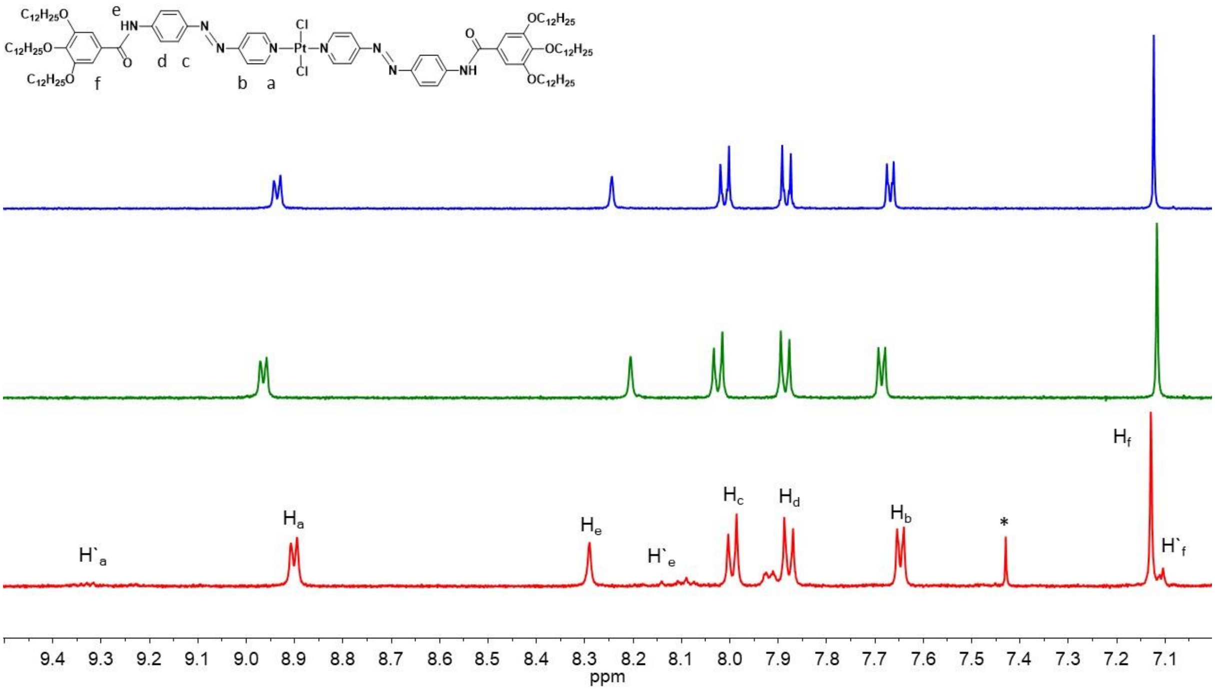

Figure S29: Time-dependent ^{1}H NMR spectra of **C_1** at 1 x 10^{-3} M showing an increase in the content of the *cis*-isomer (fresh: 0% (top), 24h: 0% (middle), 7d: 10% (bottom)) (600 MHz, DCM-d_2, 298 K).

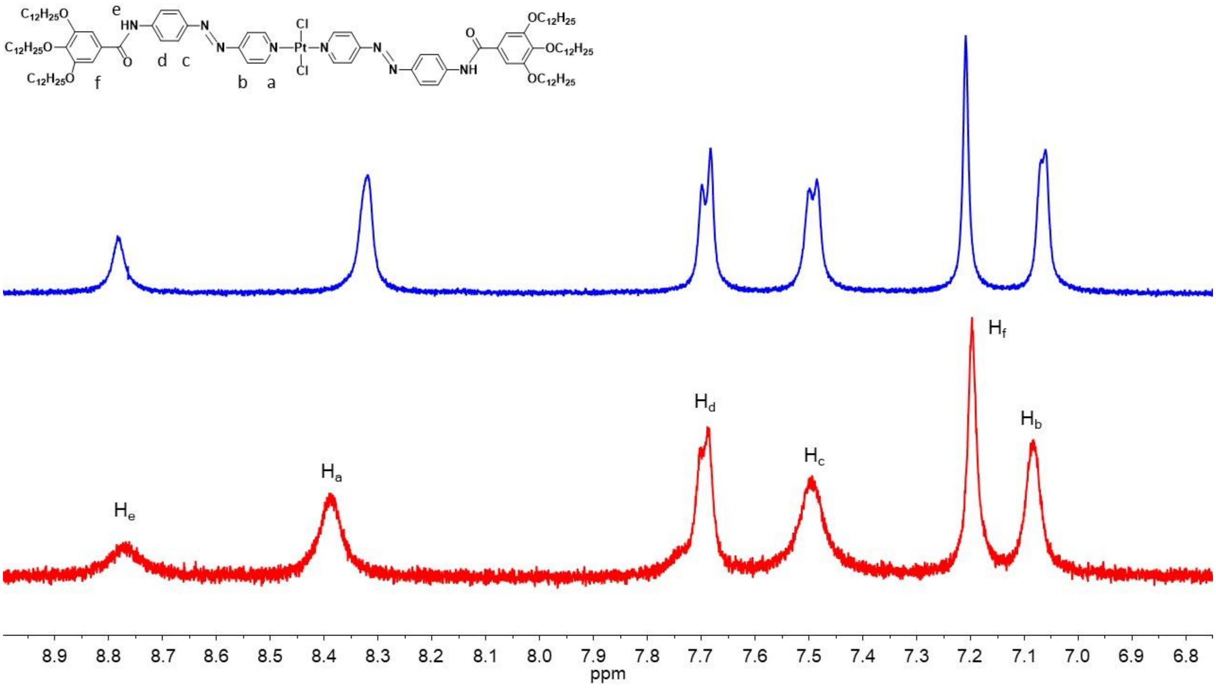

Figure S30: Time-dependent ^{1}H NMR spectra of **C₁** at 1 x 10^{-3} M showing no increase in the content of the *cis*-isomer (fresh: 0% (top), 7d: 0% (bottom)) (600 MHz, MCH-d_{14}, 363 K).

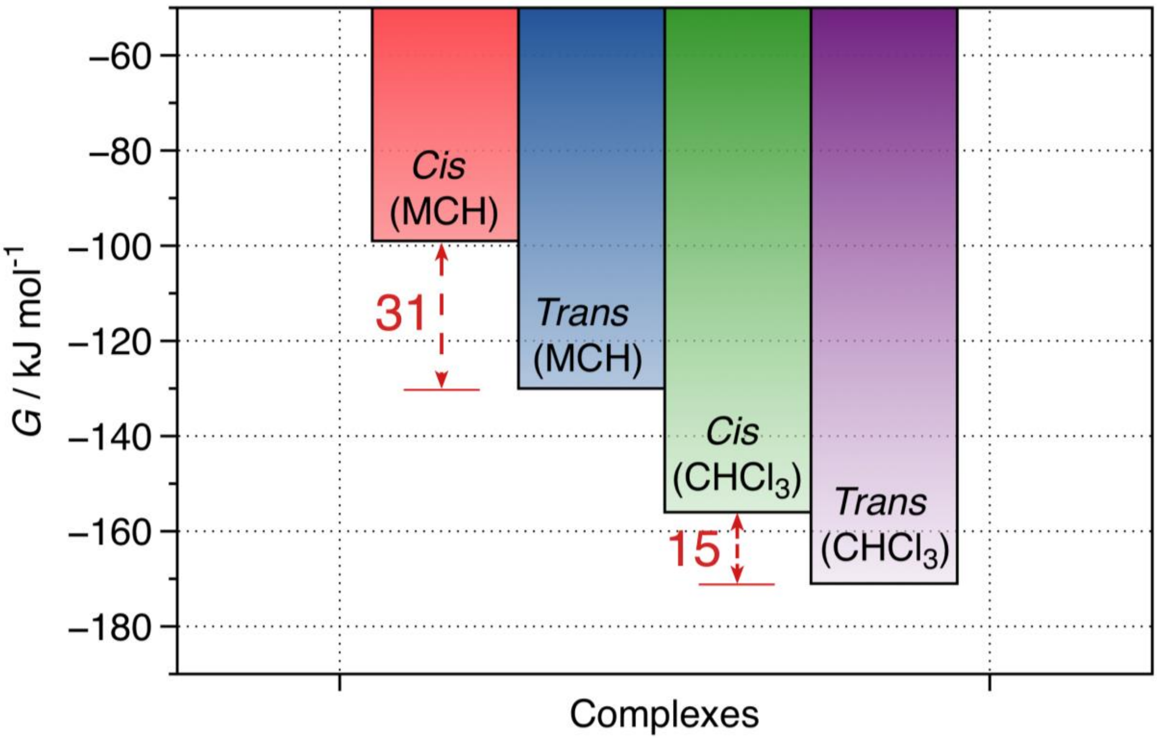

Figure S31: Gibbs energy of monomers of **C_1** optimized in different solvents assuming different coordination isomerism (Cis or Trans). The DFT calculations were carried out at the 6-31G*/LANL2DZ level and PBE0 functional with implicit solvent considered via the polarisable continuum model. As evident from the calculations, the Δ*G* dramatically decreases with increasing solvent polarity, in agreement with our experimental data

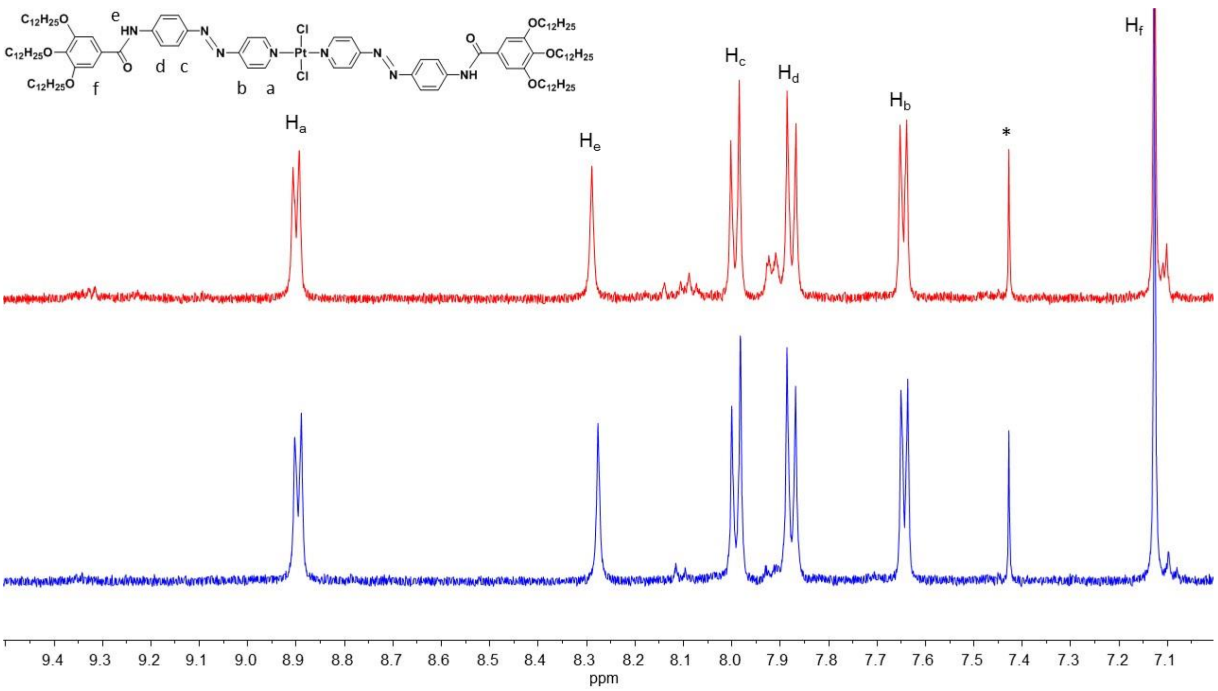

Figure S32: ^{1}H NMR spectra of a mixture of both isomers of **C₁** at 1 x 10^{-3} M (600 MHz, DCM-d_2, 298 K) before and after irradiation for 60 min. with a LED at 365 nm.

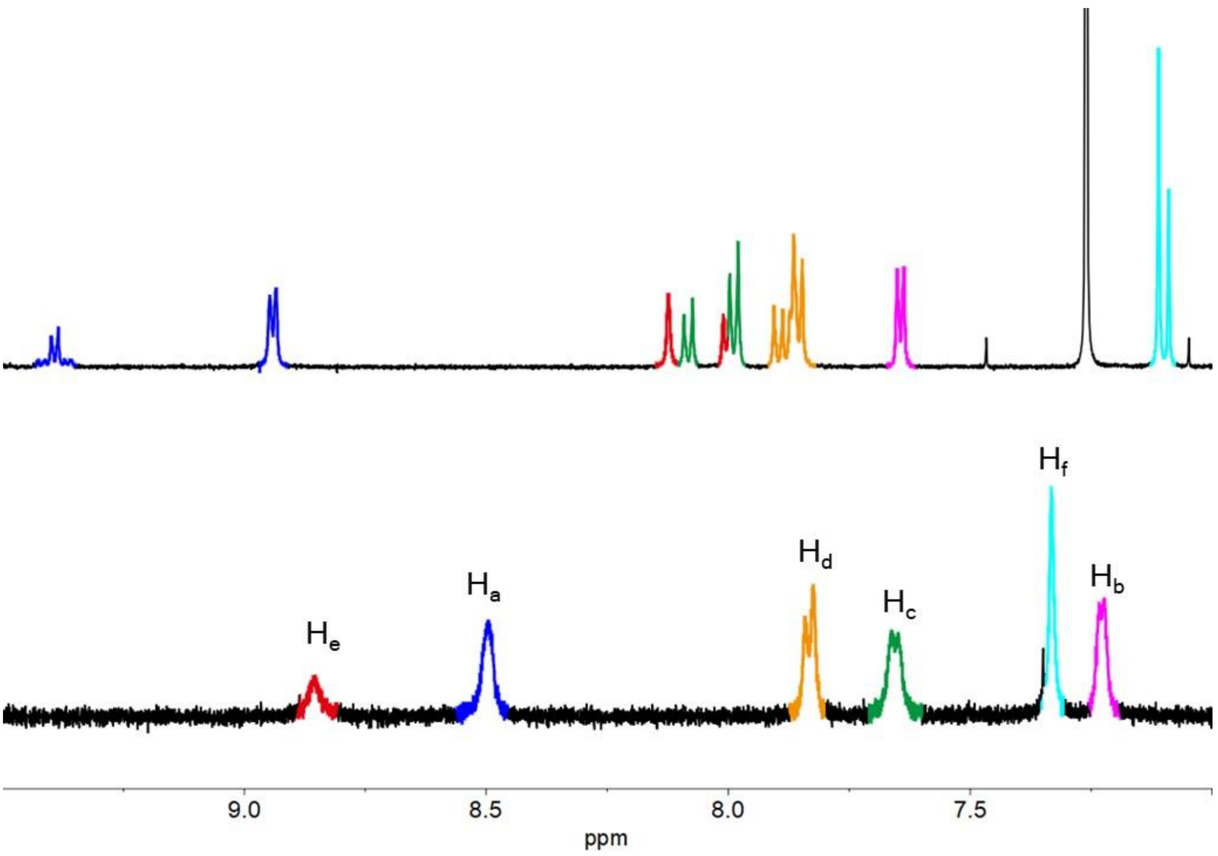

Figure S33: ^{1}H NMR spectra of a mixture of both isomers of **C_1** at 1 x 10^{-3} M (600 MHz, $CDCl_3$, 298 K) and after fast solvent removal and re-dissolution in MCH-d_{14} (600 MHz, MCH-d_{14}, 363 K).

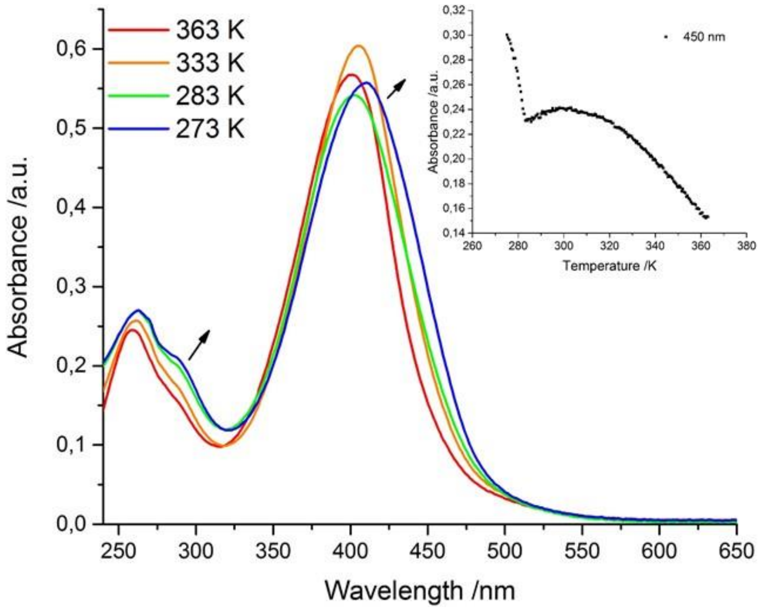

Figure S34: VT UV-Vis spectra of both isomers of **C_1** in MCH with the corresponding cooling curve presented as an inset at 1 x 10^{-5} M.

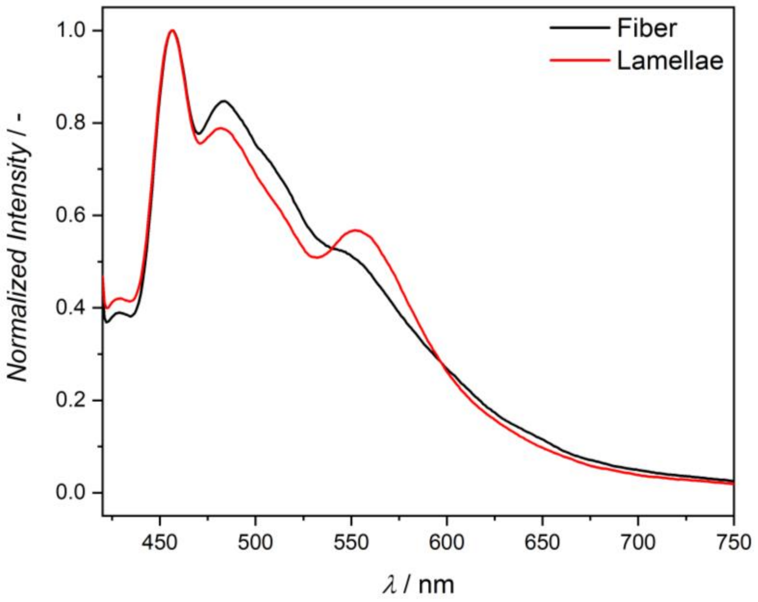

Figure S35: Emission spectra of the 1D-fibers formed from cooling a solution of pure *trans*-**C_1** (measured at 273 K in MCH with a concentration of 3 x 10^{-5} M) and the 2D-lamellae formed from cooling a mixture of both isomers (measured at 273 K in MCH with a concentration of 3 x 10^{-5} M) with an excitation wavelength of λ = 400 nm. Both aggregated species show the highest emission at 455 nm with shoulders around 480 and 550 nm confirming the aggregate formation. With the shoulder at 550 nm being more pronounced by the formation of 2D-lamellae.

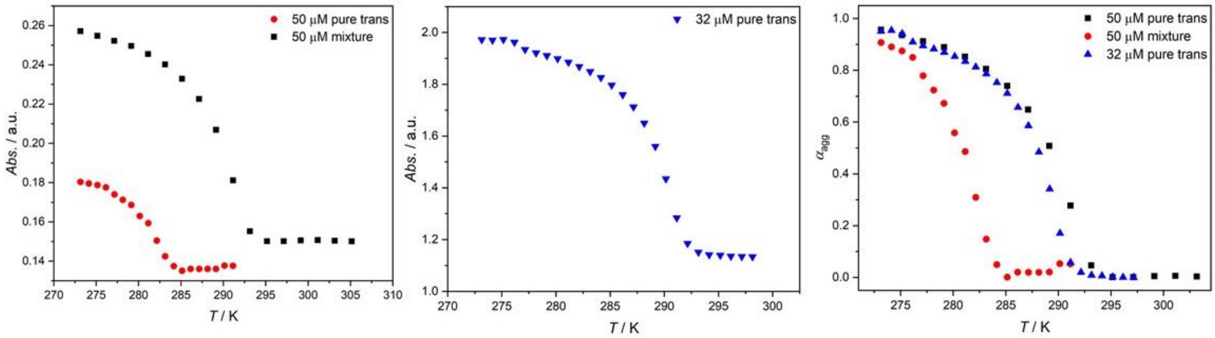

Figure S36: Plot of Abs_{450} *vs*. *T* for pure *trans*-**C**$_1$ and the 33% *cis* – 67% *trans* mixture of both isomers in MCH at 5 x 10^{-5} M (left) and for pure *trans*-**C**$_1$ in MCH at 3.2 x 10^{-5} M (middle). Right: Plot of α_{agg} *vs*. *T* for pure *trans*-**C**$_1$ and the 33% *cis* – 67% *trans* mixture of both isomers in MCH at 5 x 10^{-5} M and for pure *trans*-**C**$_1$ in MCH at 3.2 x 10^{-5} M derived from measuring the spectral changes at 450 nm to investigate the aggregation of both species (right) obtained from applying equation 1 and 2. The observed curves indicate that the *cis*-isomer acts as a dormant species for the aggregation of *trans*-**C**$_1$.

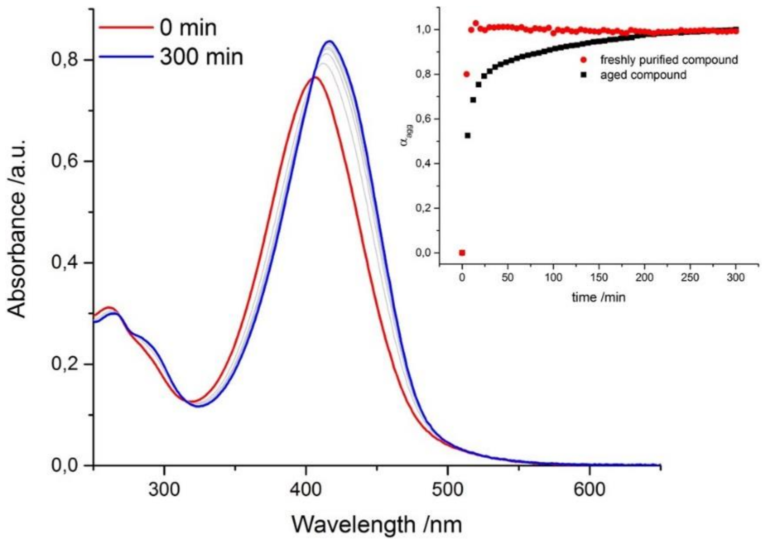

Figure S37: Time-dependent UV-Vis spectra of **C**$_1$ in MCH at 1 x 10^{-5} M after quenching from 363 to 273 K. Inset: comparison between α_{agg} monitored at 450 nm obtained from a measurement with a freshly purified compound and after aging in $CHCl_3$ for 24 h at 298 K.

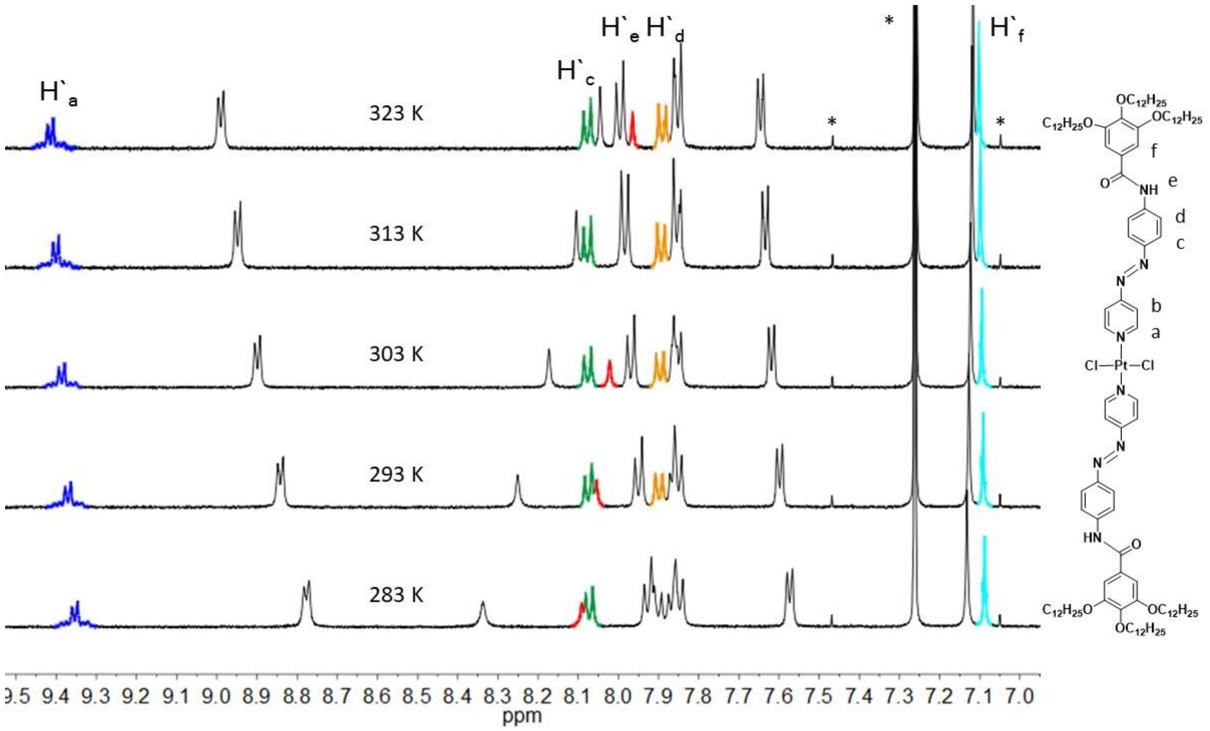

Figure S38: VT ^{1}H NMR studies of a mixture of 33% *cis* and 67% *trans*- $\mathbf{C_1}$ at 2.5 x 10^{-3} M ((600 MHz, $CDCl_3$) between 323 and 283 K. For clarity reasons only the signals of the *cis*-isomer have been colored.

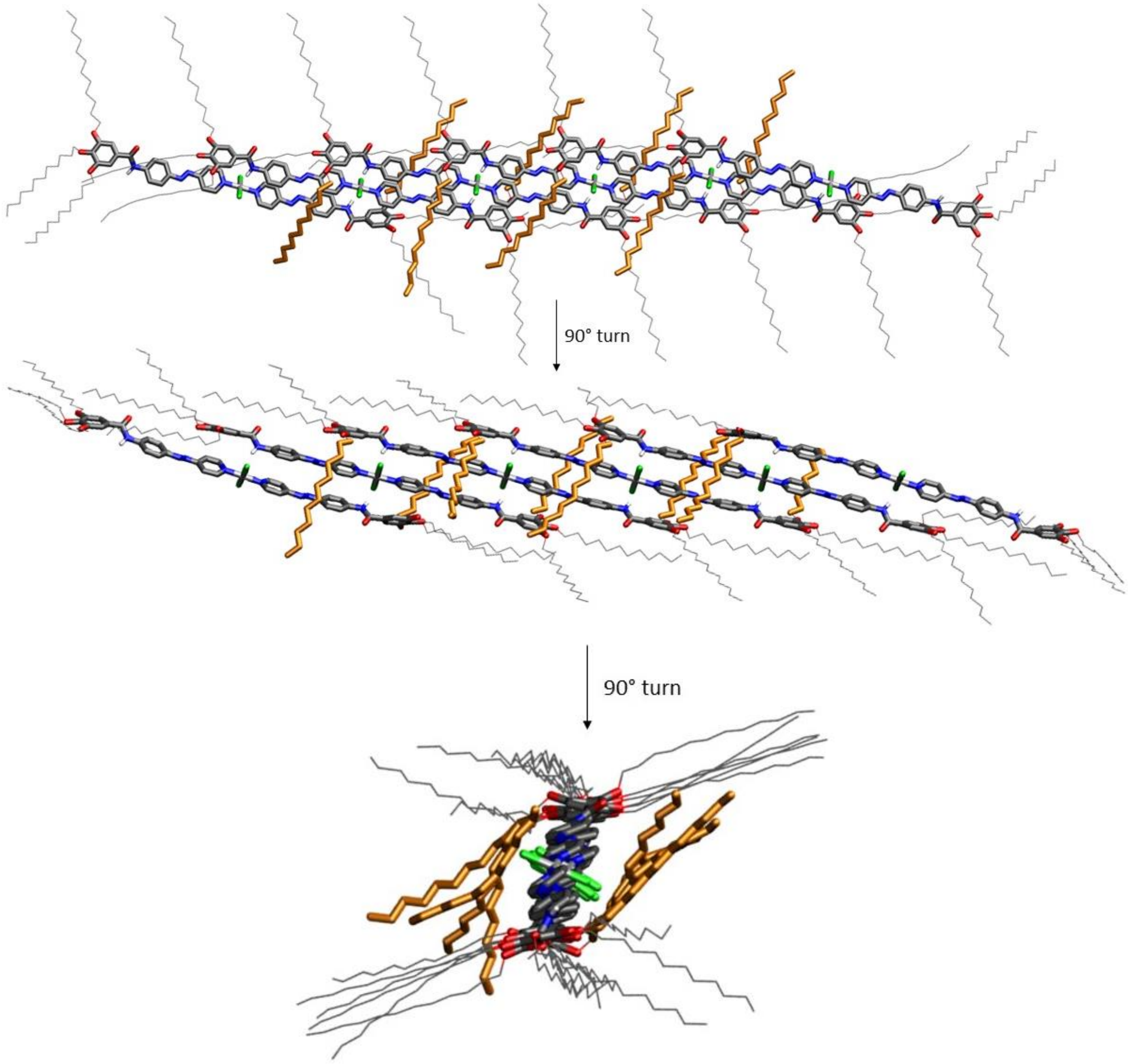

Figure S39: Dispersion-corrected PM6 model simulation in vacuum for a hexamer containing only the *trans* isomer viewed from different angles. Final heat of formation ΔH_f = -9283 kJ/mol.

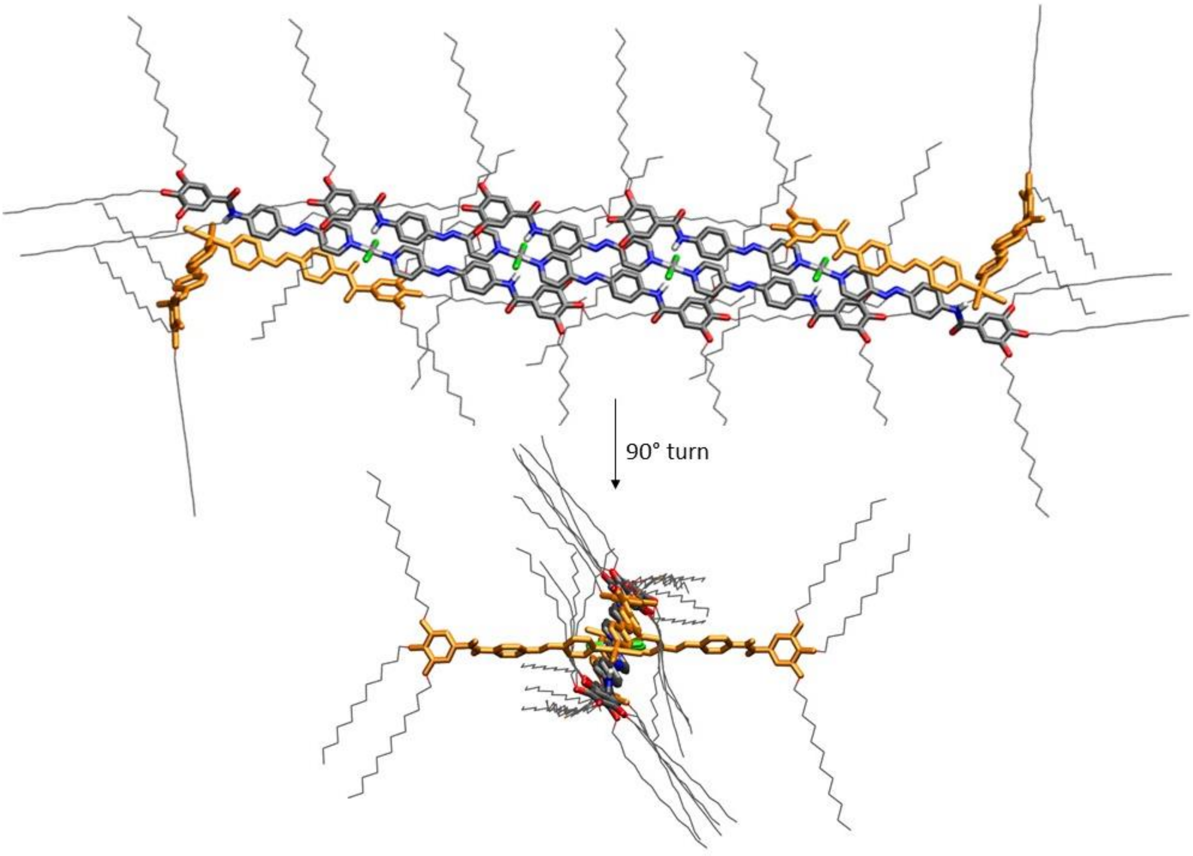

Figure S40: Dispersion-corrected PM6 model simulation in vacuum for a hexamer containing four *trans* isomers in the middle and capped by two *cis* isomers on either side from different angles. Final heat of formation ΔH_f = -9030 kJ/mol.

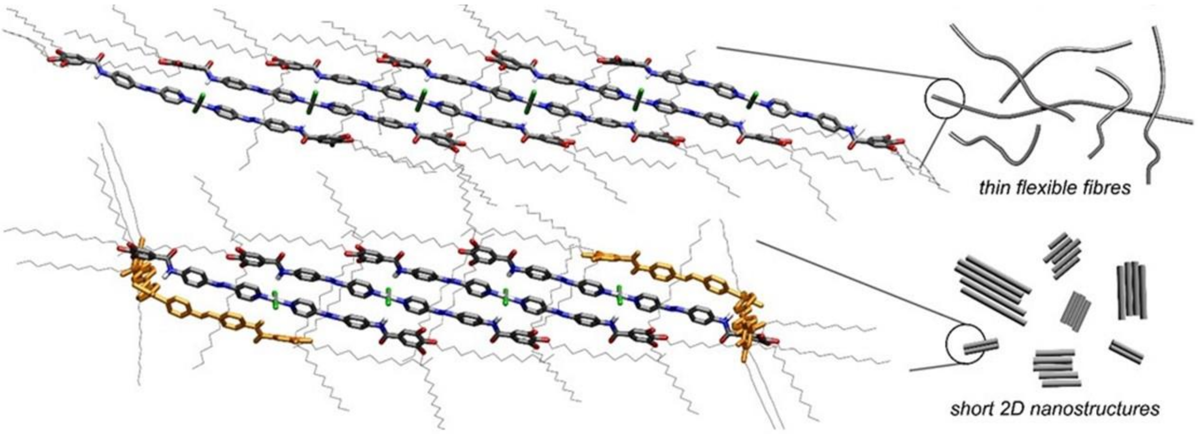

Figure S41: Dispersion-corrected PM6 model simulation in vacuum for a hexamer containing six *trans* isomers (top) and for a hexamer containing four *trans* isomers in the middle and capped by two *cis* isomers on either side (bottom) with their corresponding supramolecular structure depicted on the right side.

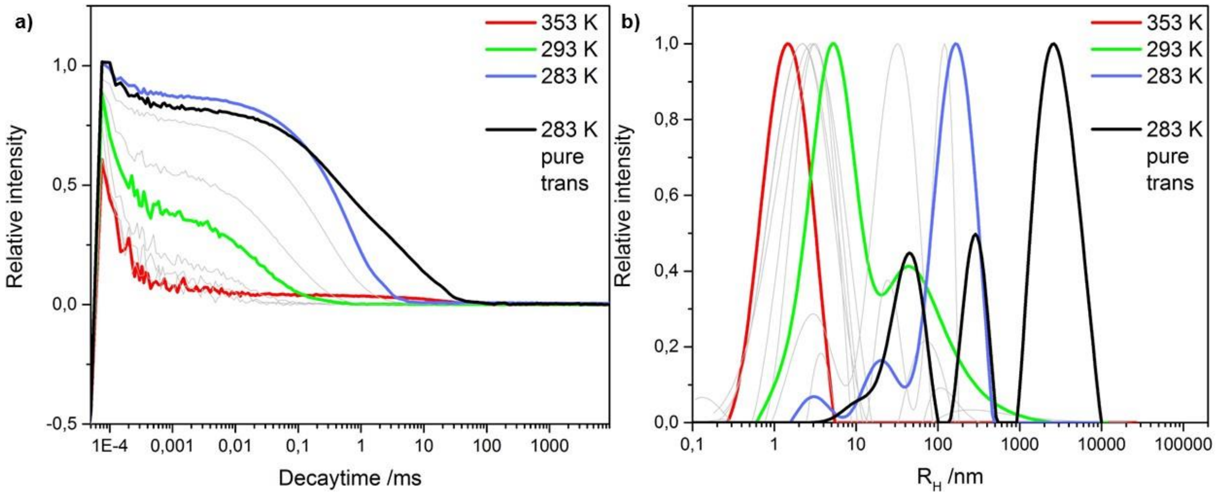

Figure S42: VT DLS studies of a mixture of both isomers (33% cis; 67% trans) of **C_1** in MCH at 2 x 10^{-4} M and comparison with the results obtained for the freshly purified complex.

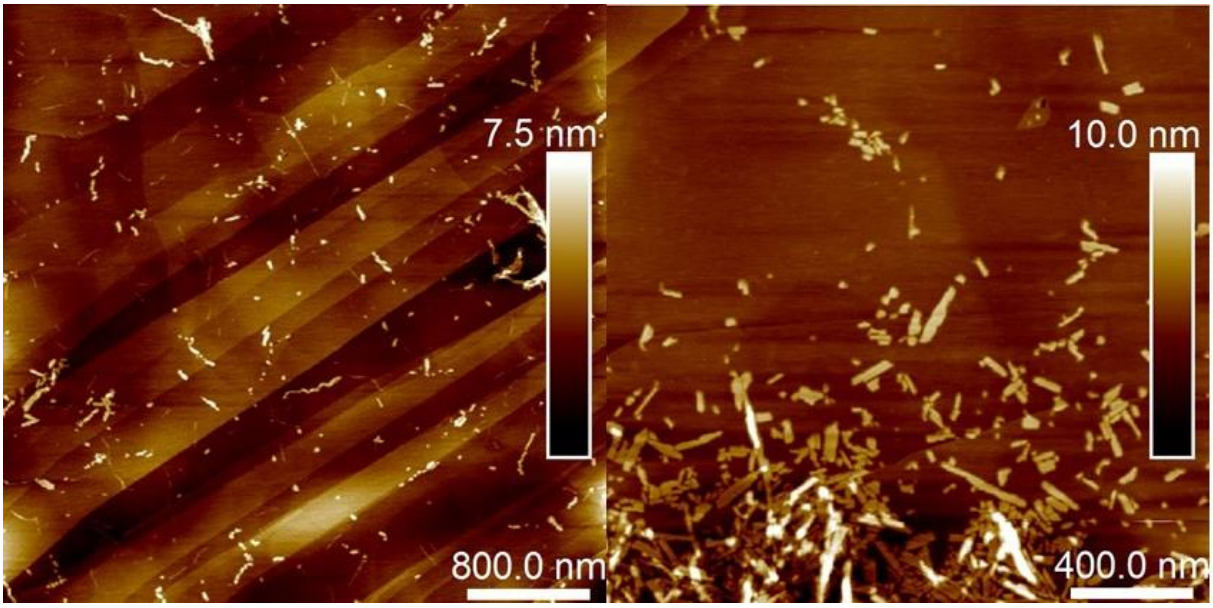

Figure S43: AFM height image of aggregates formed by a mixture of the two isomers of **C_1** in MCH at 2 x 10^{-5} M after cooling a solution from 363 to 283 K with a cooling rate of 0.2 K/min.

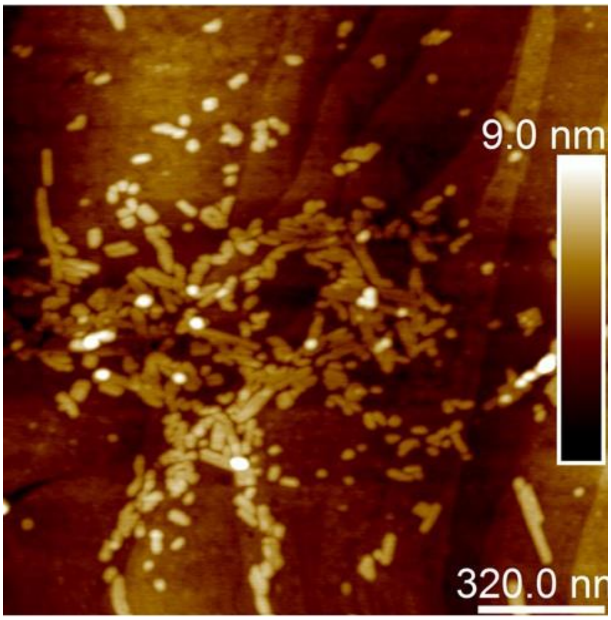

Figure S44: AFM height images of aggregates formed by a mixture of the two isomers of **C_1** in MCH after cooling a solution from 363 to 273 K with a cooling rate of 0.2 K/min at 2 x 10^{-5} M.

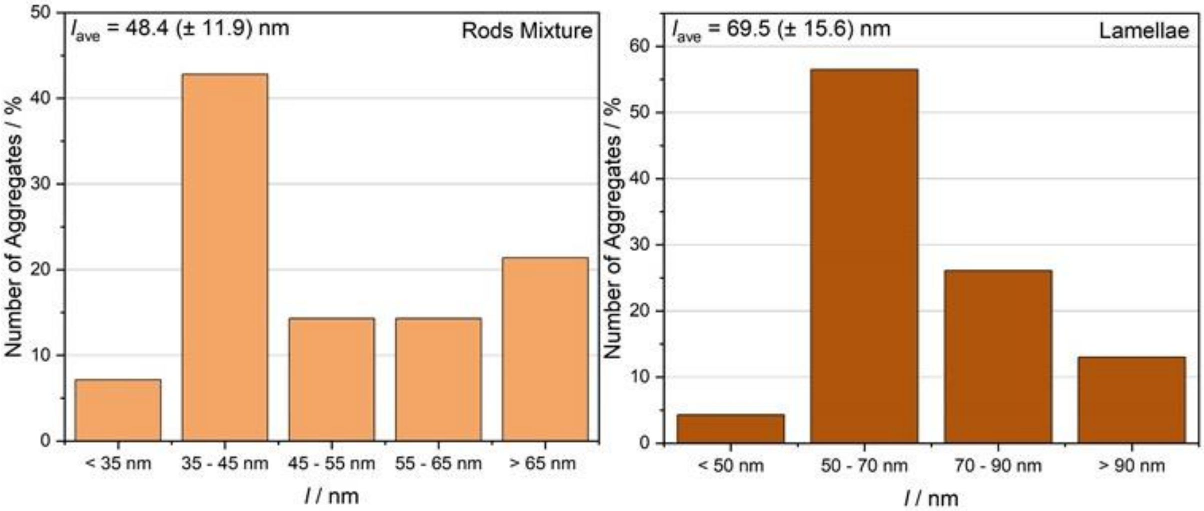

Figure S45: Size distribution of the polymer lengths as calculated from AFM images of the aggregates formed by a mixture of both isomers in MCH after cooling a solution from 363 to 283 (left) and 273 K (right) with a cooling rate of 0.2 K/min at 5 x 10^{-5} M.

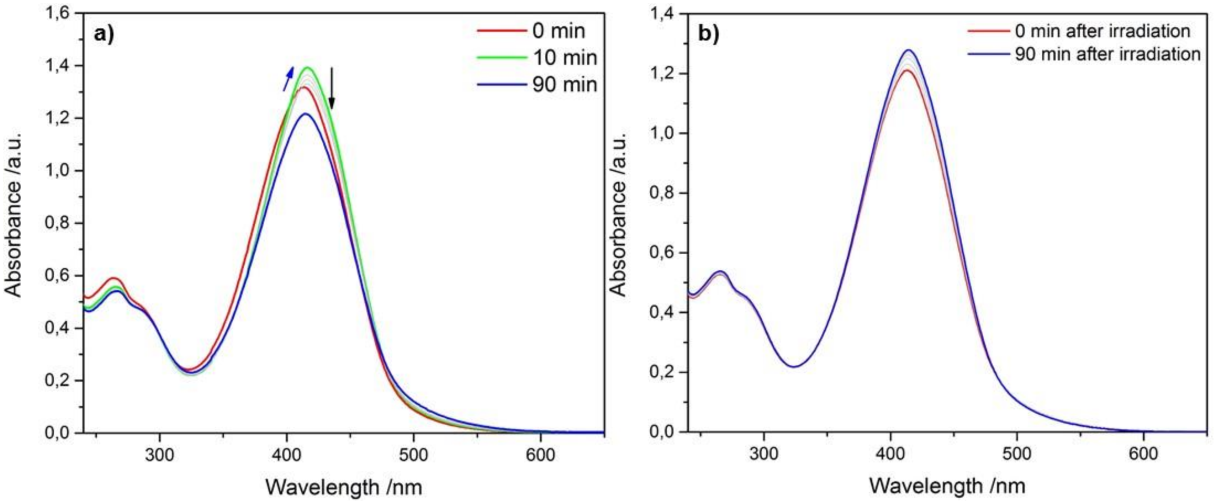

Figure S46: a) Time-dependent UV-Vis spectra of a mixture of the two isomers of **C_1** in MCH (c = 2 x 10^{-4} M, 273 K, l = 1 mm) during irradiation. The blue arrow indicates the spectral change during the first 10 min. of irradiation whereas the black arrow depicts the spectral changes after 10 min. b) time-dependent UV-Vis spectra after the irradiation process was terminated.

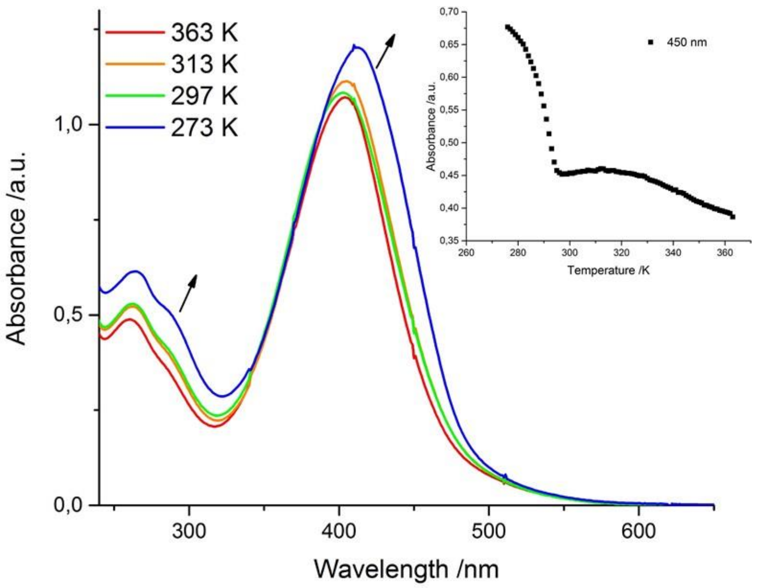

Figure S47: VT UV-Vis studies of a mixture of the two isomers of **C_1** in MCH after irradiating the sample at 363 K with a LED emitting at 365 nm for 30 min. Inset: Cooling curve obtained by monitoring at 450 nm. Arrows indicate the spectral changes below 297 K.

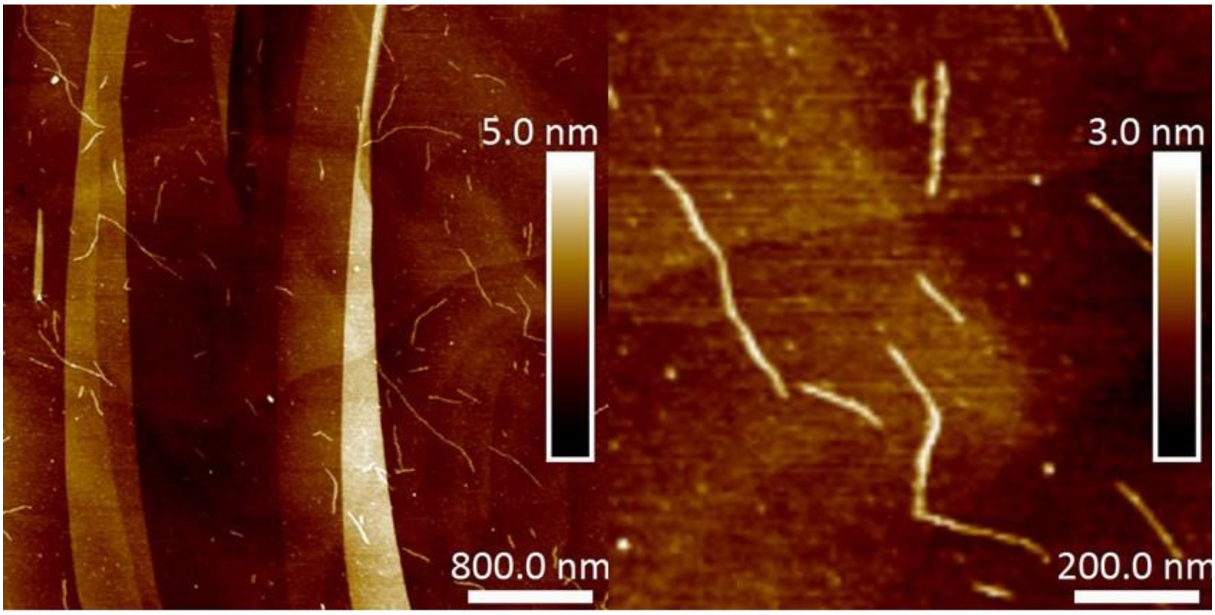

Figure S48: AFM height images of **C_1** in MCH (T = 273 K, c = 5 x 10^{-5} M) after irradiation of a mixture of both isomers at 363 K for 5 min with a UV-LED and subsequent cooling with a rate of 0.2 K/min from 363 to 273 K.

6. Literature

[1] K. K. Kartha, N. K. Allampally, A. T. Politi, D. D. Prabhu, H. Ouchi, R. Q. Albuquerque, S. Yagai, G. Fernández, *Chem. Sci.* **2019**, *10*, 752.

[2] H. M. M. ten Eikelder, A. J. Markvoort, T. F. A. de Greef, P. A. J. Hilbers, *The journal of physical chemistry. B* **2012**, *116*, 5291.

[3] a) G. Auböck, M. Chergui, *Nat. Chem.* **2015**, *7*, 629-633; b) M. E. Moustafa, M. S. McCready, P. D. Boyle, R. J. Puddephatt, *Dalton Trans.* **2017**, *46*, 8405; c) E. Borré, J.-F. Stumbé, S. Bellemin-Laponnaz, M. Mauro, *Chem Commun.* **2017**, *53*, 8344.
